# Fluctuating selection on migrant adaptive sodium transporter alleles in coastal *Arabidopsis thaliana*

**DOI:** 10.1073/pnas.1816964115

**Published:** 2018-12-07

**Authors:** Silvia Busoms, Pirita Paajanen, Sarah Marburger, Sian Bray, Xin-Yuan Huang, Charlotte Poschenrieder, Levi Yant, David E. Salt

**Affiliations:** ^a^Department of Cell and Developmental Biology, John Innes Centre, NR4 7UH Norwich, United Kingdom;; ^b^Department of Plant Physiology, Universitat Autònoma de Barcelona, Barcelona 08193, Spain;; ^c^State Key Laboratory of Crop Genetics and Germplasm Enhancement, Nanjing Agricultural University, 210095 Nanjing, China;; ^d^College of Resources and Environmental Sciences, Nanjing Agricultural University, 210095 Nanjing, China;; ^e^School of Life Sciences, University of Nottingham, Nottingham NG7 2RD, United Kingdom;; ^f^Division of Plant and Crop Sciences, School of Biosciences, University of Nottingham, Nottingham NG7 2RD, United Kingdom

**Keywords:** saline adaptation, HKT1, fluctuating selection, population genetics, evolution

## Abstract

Stressors such as soil salinity and dehydration are major constraints on plant growth, causing worldwide crop losses. Compounding these insults, increasing climate volatility requires adaptation to fluctuating conditions. Salinity stress responses are relatively well understood in *Arabidopsis thaliana*, making this system suited for the rapid molecular dissection of evolutionary mechanisms. In a large-scale genomic analysis of Catalonian *A. thaliana*, we resequenced 77 individuals from multiple salinity gradients along the coast and integrated these data with 1,135 worldwide *A. thaliana* genomes for a detailed understanding of the demographic and evolutionary dynamics of naturally evolved salinity tolerance. This revealed that Catalonian varieties adapted to highly fluctuating soil salinity are not Iberian relicts but instead have immigrated to this region more recently. De novo genome assembly of three allelic variants of the high-affinity K^+^ transporter (*HKT1;1*) locus resolved structural variation between functionally distinct alleles undergoing fluctuating selection in response to seasonal changes in soil salinity. Plants harboring alleles responsible for low root expression of *HKT1;1* and consequently high leaf sodium (*HKT1;1*^*HLS*^) were migrants that have moved specifically into areas where soil sodium levels fluctuate widely due to geography and rainfall variation. We demonstrate that the proportion of plants harboring *HKT1;1*^*HLS*^ alleles correlates with soil sodium level over time, *HKT1;1*^*HLS*^-harboring plants are better adapted to intermediate levels of salinity, and the *HKT1;1*^*HLS*^ allele clusters with high-sodium accumulator accessions worldwide. Together, our evidence suggests that *HKT1;1* is under fluctuating selection in response to climate volatility and is a worldwide determinant in adaptation to saline conditions.

## Significance

The natural landscape contains a highly heterogeneous array of environments that drive the adaptive differentiation of populations, including adaptation to elevated salinity. Our research emphasizes an integrated genetic, physiological, and ecological approach to understand the role of naturally evolved high-affinity K^+^ transporter (*HKT1;1*) allelic variants in the adaptation of *Arabidopsis thaliana* populations to fluctuating salinity dynamics in nature. This information not only provides a case study fruitfully taking identification of natural variants through population demographic dynamics to molecular function but also is valuable for improving the sustainability of crop yields as the stress from salinity escalates due to increasing population pressures and global climate change.

Stressors such as soil salinity and dehydration are major factors limiting plant growth and productivity, causing yield losses in crops around the world. High soil NaCl concentrations decrease the ability of roots to extract water, and high concentrations in plant tissues are toxic in most species. Because NaCl is widespread in the environment, plants have evolved diverse mechanisms to regulate NaCl accumulation and to select against it in favor of other nutrient ions commonly present in lower concentrations (1).

While *Arabidopsis thaliana* is a relatively salt-sensitive species (2), there are marked exceptions, especially among coastal populations (3, 4). For example, two accessions (Ts-1 and Tsu-1) accumulate much higher Na^+^ levels in their leaves while still maintaining elevated salinity tolerance. This suggests that exceptional wild *A. thaliana* accessions behave as salt-tolerant varieties, exhibiting the capacity to accumulate elevated leaf Na^+^, potentially for osmotic balance. We previously found clear evidence for local adaptation in Catalonian *A. thaliana* populations in contrasting coastal and inland environments (5). A marked cline in leaf and soil sodium content declined from the coast to inland, with coastal plants tolerating elevated salinity much better than inland plants when grown in common conditions. We demonstrated that elevated sodium in coastal soils acts as an agent of divergent selection, favoring alleles that allow the plants to tolerate the elevated soil sodium (5). Using genome-wide association studies (GWAS) and complementation tests (6), we determined that different naturally occurring alleles of the Na^+^ transporter *HKT1;1* (high-affinity potassium transporter) control leaf Na^+^ across a broad range of concentrations. Accessions with *HKT1;1* alleles driving high leaf sodium (HLS), here called “*HKT1;1^HLS^*,” such as Ts-1 and Tsu-1, are mainly distributed in coastal regions or in exceptional inland regions with elevated salinity (6). This association strongly suggests that soil salinity levels drive the observed landscape distribution of the *HKT1;1*^*HLS*^ allele. Supporting this, An et al. (4) recently established genetically that the *HKT1;1*^*HLS*^ allele confers the elevated salinity tolerance previously observed in the Tsu-1 accession (3).

The *HKT* family of transporters is implicated in Na^+^ unloading from xylem vessels and typically functions as K^+^/Na^+^ symporters at the plasma membrane (7–13). In the Col-0 accession *HKT1;1* is mainly expressed in roots, where it functions to retrieve Na^+^ from the xylem to reduce the transport of Na^+^ to shoots to maintain low leaf sodium (LLS); we name this allele “*HKT1;1*^*LLS*^” (9, 14–16). The *HKT1;1*-null mutation in Col-0 results in a significant alteration of Na^+^ distribution in the plant, with higher levels of Na^+^ accumulating in shoots and lower levels in roots compared with wild-type Col-0. The *HKT1;1*-null allele is also significantly more sensitive to NaCl (9, 17, 18). This contrasts with the *HKT1;1*^*HLS*^ allele in accessions such as Ts-1 and Tsu-1, which has low expression in roots and is expressed mainly in stem xylem tissue. The *HKT1;1*^*HLS*^ allele functions to limit sodium accumulation in flowers and confers elevated salinity tolerance compared with the *HKT1;1*^*LLS*^ allele when each is expressed in Col-0 (4).

We focus here on a group of coastal Catalonian *A. thaliana* populations that offer a natural laboratory to understand the evolutionary and ecological dynamics of saline adaptation. We identify seven stands of plants (demes) harboring the *HKT1;1*^*HLS*^ allele (Ts-1– and Tsu-1–like) in habitats close to, but not at, the coast. All these demes also contain plants with the *HKT1;1*^*LLS*^ (Col-0–like) allele. There is abundant evidence that the direction of selection can vary among microsites within contiguous plant populations (e.g., ref. 19). Such heterogeneity can maintain alternative alleles and aid in adaptation to fluctuating environments. We hypothesize that the presence of the *HKT1;1*^*HLS*^ allele in these demes confers salinity tolerance through limiting the accumulation of Na in flowers (4) and that both alleles are maintained because the soil salinity fluctuates temporally. We suggest that the widespread within-population cooccurrence of the *HKT1;1*^*HLS*^ and *HKT1;1*^*LLS*^ alleles represents a case of fluctuating selection in response to dynamically changing seasonal saline conditions present specifically in intermediate-coastal environments. To understand the evolutionary and demographic dynamics of this system, we performed a large-scale population study, enabling an analysis of worldwide *HKT1*;*1*-harboring genomes along with the ecological and physiological implications. We present a detailed locus description of the major *HKT1;1* haplotypes in these Catalonian demes as well as worldwide. These data confirm that variation at the *HKT1;1* locus is a major global determinant of saline adaptation and suggest that the rapidly varying within-deme dynamics of the *HKT1;1* alleles allows nimble adaptation to highly variable seasonal soil salinity.

## Results and Discussion

*A. thaliana* has been widely used in studies of plant genetic diversity, adaptation, and biogeography (see refs. 20 and 21 for reviews). The rapid Holocene expansion of *A. thaliana* into northern Europe from Asian and Mediterranean refugia suggests a high potential to disperse to diverse environments (22). In particular, we have strong evidence for local adaptation to the coast in wild demes of *A. thaliana* from Catalonia (Spain), where soil salinity is a major selective force (5).

We previously observed that the *HKT1;1*^*HLS*^ allele occurs primarily in plants located in coastal environments, including in Catalonia, and based on this distribution, we proposed, that it may be involved in enhanced salinity tolerance (6). The recent molecular genetic discovery that *HKT1;1*^*HLS*^ confers enhanced tolerance to salinity through exclusion of sodium from reproductive tissues strongly supports its role in adaptation to coastal environments (4). To further explore the role of *HKT1;1*^*HLS*^ in local adaptation to coastal saline habitats, we undertook a multiyear study in the field at sites in northeastern Catalonia where coastally adapted *A. thaliana* demes exist (5).

### Distribution, Assembly, and Characterization of the *HKT1;1* Genomic Locus.

**Fig. 1.**
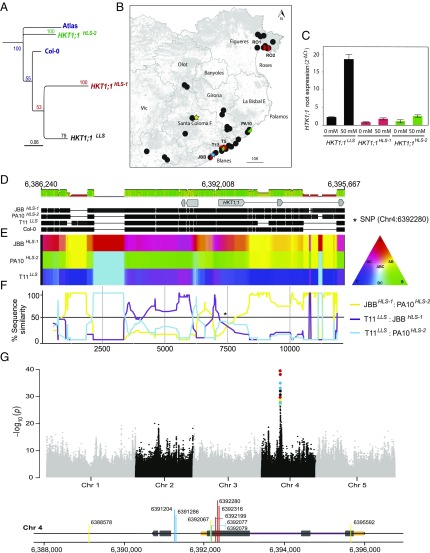
Geographic location, genomic structural variation, and functional impact of major *HKT1;1* variants. (*A*) Phylogenetic consensus tree of the *HKT1;1* locus from 77 Catalonian *A. thaliana* individuals grouped as *HKT1;1*^*LLS*^ (black), *HKT1;1*^*HLS-1*^ (red), and *HKT1;1*^*HLS-2*^ (green), with the Col-0 reference and an African relict (Atlas) used as an outgroup. (*B*) Map of the 36 *A. thaliana* demes in northeastern Catalonia (black dots: demes harboring *HKT1;1*^*LLS*^; black/red dots: demes with a mix of plants carrying either *HKT1;1*^*LLS*^ or *HKT1;1*^*HLS-1*^; black/green dots: demes with a mix of plants carrying either *HKT1;1*^*LLS*^ or *HKT1;1*^*HLS-2*^) and the two field sites chosen to perform reciprocal transplant experiments at BLA (blue star) and SCF (green star). (*C*) Transcript expression of *HKT1;1* in roots of triplicate biological replicates following exposure to 0 mM or 50 mM NaCl in hydroponics for 2 wk. Data represent the mean ± SE. (*D*) Sequence alignment of the de novo-assembled *HKT1;1* locus for *HKT1;1*^*HLS-1*^ (JBB), *HKT1;1*^*HLS-2*^ (PA10), *HKT1;1*^*LLS*^ (T11), and Col-0. *HKT1;1* CDS (introns and exons) are indicated by thin and thick gray lines, respectively. Sequence similarity is represented by a color scale on the bar above the sequence alignment: green, 100%; khaki, 75%; red, 50%. (*E*) Sequence similarity at the same region among the three *HKT1;1* versions is visualized using a color triangle. Areas where two rows show the same color indicate localized high similarity between sequences. (*F*) Linear plot showing the proportion of SNPs shared between the three pairwise sequence comparisons. The asterisk indicates the *HKT1;1* diagnostic SNP (Chr4:6392280) position. (*G*) Manhattan plot of GWA mapping results. SNPs are plotted according to genomic position. Positions of the significant associations at *HKT1;1* are represented in the alignment below the Manhattan plot and are colored according the *HKT1;1* allelic variants they discriminate (*SI Appendix*, Table S3): red, only *HKT1;1*^*HLS-1*^; blue, only *HKT;1*^*LLS*^; yellow, both *HKT1;1*^*HLS-1*^ *and HKT1;1*^*HLS-2*^.

In March of 2013, 2014, and 2015, we sampled 36 *A. thaliana* demes from northeastern Catalonia and genotyped them using an established marker that differentiates *HKT1;1*^*LLS*^ and *HKT1;1*^*HLS*^ alleles [SNP at Chr4:6392280: cytosine (C) linked to *HKT1;1*^*LLS*^ and thymine (T) linked to *HKT1;1*^*HLS*^] (6). In addition, in 2015 we collected and individually sequenced the genomes of 74 individuals from 19 demes (Dataset S1) to >25× genome-wide coverage to generate very-high-density genomic markers. Focusing on the *HKT1;1* locus structure, a phylogenetic analysis rooted by an African *A. thaliana* relict individual shows three clearly differentiated groups by different clustering methods (Fig. 1*A* and *SI Appendix*, Fig. S1). We have named these “*HKT1;1*^*LLS*^” (high root expression, marked with a C at diagnostic SNP), “*HKT1;1*^*HLS-1*^” (low root expression, marked with T at diagnostic SNP), and the newly identified “*HKT1;1*^*HLS-2*^” (low root expression, marked with a C at the diagnostic SNP).

We identified 29 demes with all plants homozygous for *HKT1;1*^*LLS*^, four demes with a mixture of plants with either the *HKT1;1*^*LLS*^ or *HKT1;1*^*HLS-1*^ alleles, one deme (PA10) with a mixture of *HKT1;1*^*LLS*^ and *HKT1;1*^*HLS-2*^ plants, and one deme (T13) with a mixture of all three *HKT1;1* alleles (Fig. 1*B* and Dataset S1). Strikingly, despite low levels of diversity (23) in this overwhelmingly selfing species, demes containing only the *HKT1;1*^*HLS*^ alleles were never observed; we always found *HKT1;1*^*HLS*^ plants in the presence of *HKT1;1*^*LLS*^ plants. Importantly, despite this consistent within-population diversity, we never identified a plant heterozygous for these alleles.

We performed an expression analysis of *HKT1;1* across three selected accessions: T11, which represents the *HKT1;1*^*LLS*^ allele; JBB, which represents the *HKT1;1*^*HLS-1*^ allele; and PA10, which represents the *HKT1;1*^*HLS-2*^ allele. As expected, *HKT1;1* expression in roots of JBB (with a T marking the *HKT1;1*^*HLS*^ allele) was low. However, even though both T11 and PA10 accessions had a C at the diagnostic SNP, we observed the expected high root expression of *HKT1;1* only in T11. *HKT1;1* expression in roots of PA10 was as low as that observed for JBB (Fig. 1*C*). Thus, based on expression levels in roots, we have identified an *HKT1;1* allele that, although it harbors the diagnostic SNP marking the *HKT1;1*^*LLS*^ allele (asterisk in Fig. 1*F*), actually exhibits low root expression as the *HKT1;1*^*HLS-1*^ allele.

To gain a high-resolution picture of each *HKT1;1* allele, we performed de novo genome assemblies with linked reads generated with the 10× Genomics Chromium platform and Supernova assembler (24) in plants harboring each *HKT1;1* allele type (T11^*LLS*^, JBB^*HLS-1*^, and PA10^*HLS-2*^), allowing us to resolve these three *HKT1;1* alleles found within our Catalonian demes (Fig. 1 *D*–*F*). The *HKT1;1* locus shows clear peaks and troughs in sequence similarity between each of the three classes (Fig. 1 *E* and *F*), reflecting runs of SNP similarity and also shared differences in structural variation between types. Both the *HKT1;1*^*HLS-1*^ and *HKT1;1*^*HLS-2*^ alleles with reduced root expression exhibit conspicuous structural variation at the locus relative to *HKT1;1*^*LLS*^ with high expression in roots. Both *HKT1;1*^*HLS-1*^ and *HKT1;1*^*HLS-2*^ feature a pronounced 700-bp deletion in the promoter as well as a 1.2-kb insertion in the second intron that may be responsible for the observed low *HKT1;1* expression in roots (Fig. 1*C*). The promoter deletion we observe was first identified by Rus et al. (3) in the Ts-1 and Tsu-1 accessions. The second intron insertion we have identified has 80% pairwise identity in *HKT1;1*^*HLS-1*^ and *HKT1;1*^*HLS-2*^ and shows high homology to a transposable element (TE) in the TAIR10 Col-0 reference, AT4G37570 (the pseudogene of putative DNA methyltransferase). Intron TE insertion events have been clearly associated with reduced expression (25). Also common to both *HKT1;1*^*HLS-1*^ and *HKT1;1*^*HLS-2*^, the promoter structural variant may additionally mediate the reduced expression we observe in roots. Indeed Rus et al. (3), and Baek et al. (16) showed a recapitulation of the reduced root expression of *HKT1;1* in a transfer DNA insertional mutant within this region. However, to confirm what impact these structural changes have on the regulation of *HKT1;1* will require the development of transgenic plants containing chimeric *HKT1;1* alleles in an *hkt1;1*-null isogenic background.

In stark contrast to the conspicuous structural variation discussed above, we detected only three fixed nonsynonymous amino acid changes in our 77 de novo assembled or resequenced genomes at this locus. At the amino acid level, the *HKT1;1*^*HLS-1*^ and *HKT1;1*^*HLS-*2^ groups are differentiated from *HKT1;1*^*LLS*^ at V24L, F476L, and V492A. We therefore generated a protein structure of *HKT1;1* using homology modeling to predict any resultant functional changes (*SI Appendix*, Fig. S2 and Table S1). The structure is in good agreement with previous structural predictions (26, 27) and functional studies (4); for example, S68 has been implicated in Na^+^ specificity by site-directed mutagenesis (27) and is situated at the narrowest point of the selectivity filter in the predicted structure. None of the fixed amino acid changes is at a highly conserved site (*SI Appendix*, Fig. S3) (8). They are all situated on the outer surface of the protein and form transmembrane helices sequestered within the membrane. Both *HLS* alleles have nonpolar hydrophobic amino acids at these three sites. Consequently, these changes are highly unlikely to have implications for *HKT1;1* function. This is consistent with the electrophysiological data comparing the function of the *HKT1;1* alleles from Col-0 and Tsu-1 (comparable to *HKT1;1*^*LLS*^ and *HKT1;1*^*HLS-1*^) in *Xenopus laevis* oocytes (4). It is also consistent with evidence that differences in *HKT1;1* expression level in roots drive the variation in leaf Na accumulation (6, 3).

As our discovery of a third *HKT1;1* allele type illustrates that the Chr4:6392280 diagnostic SNP is in fact insufficient to discriminate between the three identified *HKT1;1* alleles, we consulted the “Na23 project” open-access dataset available in the AraGWAS catalog (28). The SNPs found to be significantly associated with *HKT1;1* by genome-wide association (GWA) analysis discriminate only the *HLS-1* allele (*SI Appendix*, Table S2). Fortunately, we have generated leaf ionomic profiles for the majority of the 1,135 *A. thaliana* genomes available on 1001genomes.org/ (Dataset S2). Therefore, we could perform a GWA analysis of leaf Na accumulation (including triallelic sites and accounting for background structure with a kinship random effect) to determine which SNPs discriminate each of the major *HKT1;1* alleles. Although the two most strongly associated SNPs discriminate only the *HLS-1* allele, we detect several SNPs among the very top associated SNPs that better represent the *HKT1;1* allelic variation found in this study (Fig. 1*G* and *SI Appendix*, Table S3). We highlight these associated SNPs (colored according to the *HKT1;1* allelic variants they discriminate) in the alignment below the Manhattan plot in Fig. 1*G*. Of particular interest are the single multiallelic SNPs at Chr4:6392067 (first exon), Chr4:6388578 (promoter), and Chr4:6395592 (third exon), because these polymorphic sites discriminate all three of the characterized *HKT1;1* alleles in this study (Fig. 1*G* and *SI Appendix*, Table S3).

### Functional Impact of *HKT1;1* Allelic Variation.

**Fig. 2.**
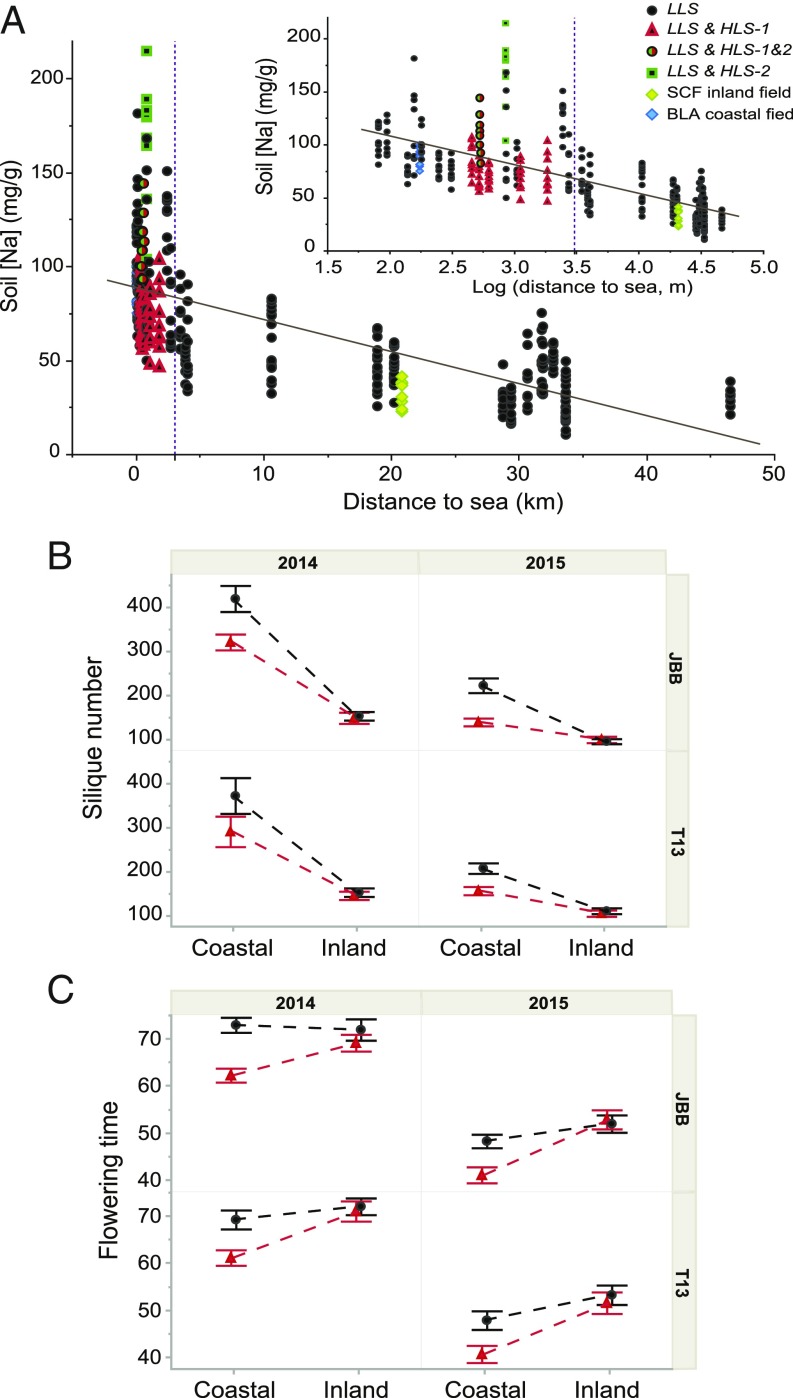
Fine-scale distribution and local adaptation of *HKT1;1* alleles. (*A*) Na concentration of triplicate soil samples from 36 *A. thaliana* demes and the two field sites (BLA and SCF) collected in March 2013, 2014, and 2015. (*Inset*) Relationship with the distance to the coast on a logarithmic scale and showing the presence of each *HKT1;1* allele. See key in figure for color and symbol code. (*B* and *C*) Fitness (*B*) and flowering time (*C*) of plants with *HKT1;1*^*LLS*^ (black dots) and plants with *HKT1;1*^*HLS*^ (red triangles) from the T13 and JBB demes cultivated at the coastal and inland fields in 2014 and 2015. Data represent the mean ± SE (*n* = 10 plants of each *HKT1;1* allele per field site, deme, and year).

All demes containing plants with *HKT1;1*^*HLS*^ (“mixed demes”) were located in coastal areas (less than 3 km from the sea), supporting the distribution of *HKT1;1*^*HLS*^ reported in ref. 6. However, focusing on the coastal areas, we observe that plants containing *HKT1;1*^*HLS*^ ^*1&2*^ occur only in an intermediate zone (500–1,500 m from the coast) where soil Na^+^ concentrations are between 50 and 150 mg/g (Fig. 2*A*).

On average, plants from coastal demes are more tolerant of elevated salinity than plants of inland origin (5), but the majority of our coastal demes lack *HKT1;1*^*HLS*^ plants. Moreover, mixed demes containing plants with *HKT1;1*^*HLS*^ are never located at sites with soils with the very highest Na^+^ concentrations, which occur within 500 m of the coast (Fig. 2*A*). These results suggest that, even though the *HKT1;1*^*HLS-1*^ allele has been established to confer enhanced salinity tolerance (4), it cannot be the sole determinant for the differential salinity tolerance we have previously observed between coastal and inland demes (5). This conclusion is supported by the fact that when plants with *HKT1;1*^*HLS-1*^ are removed from a reanalysis of our previously published common field garden experiment data, we still observe a clear signal of local adaptation (*SI Appendix*, Fig. S5 *A* and *B* and Table S4).

If *HKT1;1*^*HLS*^ is not the main driver of coastal adaptation, then several intriguing questions arise. First, why is it localized to a narrow region adjacent to, but not at, the coast? Second, what is its beneficial function in this region? Third, why does it always occur in mixed demes containing both alleles? We treat each of these questions in turn below.

To address the question of why the *HKT1;1*^*HLS*^ allele is localized to a zone adjacent to the coast with soil salinity intermediate between the coast and inland, we grew plants harboring *HKT1;1*^*LLS*^ and *HKT1;1*^*HLS-1*^ from two mixed demes in common gardens at the coast (Blanes, hereafter “BLA”) and inland (Santa Coloma de Farners, hereafter “SCF”) that we had previously used (stars in Fig. 1*B*) (5). Importantly, our BLA common garden site was located 230 m from the sea, a location where plants with the *HKT1;1*^*HLS-1*^ allele do not occur naturally. This allowed us to test the hypothesis that the *HKT1;1*^*HLS-1*^ allele is in fact maladaptive very close to the coast.

Reciprocal experiments performed in 2014 and 2015 showed that, based on silique production, plants with the *HKT1;1*^*HLS-1*^ allele from two different demes (separated by 15 km) were less fit than plants with the *HKT1;1*^*LLS*^ allele when grown directly at the coast. However, no difference was observed between plants with the two alternative alleles when grown inland (Fig. 2*B*). Further, independent of the *HKT1;1* locus, coastal demes outperformed inland demes when grown at the coast (*SI Appendix*, Fig. S5*C*). From these observations, we conclude that *HKT1;1*^*HLS-1*^ is maladaptive in soils with the highest salinity very close to the coast even when it occurs in a coastally adapted genetic background. This maladaptation very close to the coast could explain why the *HKT1;1*^*HLS-1*^ allele does not occur naturally in demes closest to the coast. However, to confirm this conclusion genetically, it will be important to perform similar studies with either near-isogenic lines that vary only at the *HKT1;1* locus or in which the genetic background is segregating independently from the *HKT1;1* locus.

Furthermore, *HKT1;1*^*HLS-1*^ plants growing at the BLA coastal common garden flowered earlier than *HKT1;1*^*LLS*^ plants cultivated at the same coastal site during the 2 y of the reciprocal transplant, but no difference in flowering time was observed at the SCF inland site (Fig. 2*C*). This difference in flowering time detected only at the BLA coastal site might be a symptom of stress (29), an adaptation to protect the plant from the increased salinity (as described in ref. 30), or a combination of both. However, this difference in flowering time could explain why we did not find any hybrid plants for the *HKT1;1* locus in these mixed demes.

It is important to note that we used the previously established SNP marker (6) to select the plants with *HKT1;1*^*LLS*^ or *HKT1;1*^*HLS-1*^ allele from the JBB and T13 demes. We have now determined that a C at this marker does not differentiate between *HKT1;1*^*LLS*^ and the newly discovered *HKT1;1*^*HLS-2*^ allele. It therefore is possible that some of the T13 plants identified as T13^*LLS*^ were actually T13^*HLS-2*^. Fortunately, we have the data for the individual leaf Na content of these plants, and, using this information, we could detect four plants in 2014 and five plants in 2015 (out of a total of 20 plants per year) that are potentially *HLS*. Reanalysis of the data excluding these potentially misattributed plants (*SI Appendix*, Fig. S5 *D* and *E*) did not substantially modify our conclusions.

**Fig. 3.**
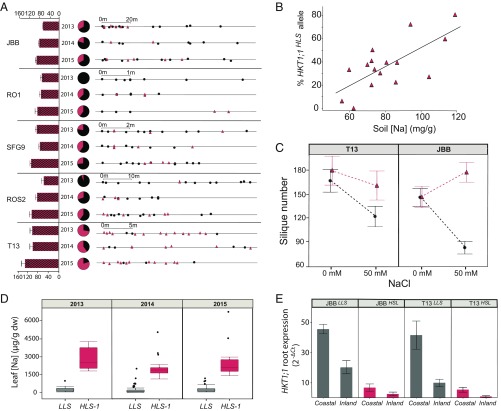
Frequency of *HKT1;1* alleles and relationship to soil Na and fitness. (*A*) *HKT1;1*^*HLS*^ allele frequencies and distributions in mixed demes over three successive years. (*Left*) The bar chart represents mean ± SE soil Na content measured in May of each year. (*Center*) Pie charts represent the ratio of plants with *HKT1;1*^*LLS*^ (black) or *HKT1;1*^*HLS*^ (red). (*Right*) *S*cales show the linear distance of each plant with respect to the first genotyped plant of each site. Black dots, *HKT1;1*^*LLS*^ plants; red triangles, *HKT1;1*^*HLS*^ plants. (*B*) Correlation between frequency of the plants with the *HKT1;1*^*HLS*^ in each deme and soil Na content over 3 y. (*C*) Fitness (number of siliques produced) of plants with the *HKT1;1*^*LLS*^ (black dots) and plants with the *HKT1;1*^*HLS*^ (red triangles) alleles from the T13 and JBB demes cultivated in potting mix soil under controlled conditions and irrigated with 0 or 50 mM NaCl. Data represent the mean ± SE (*n* = 10 plants per *HKT1;1* allele version per treatment). (*D*) Na content in leaves (dw, dry weight) from plants with the *HKT1;1*^*LLS*^ (black boxes) and plants with the *HKT1;1*^*HLS*^ (red boxes) alleles collected from their natural habitats in 2013 (*n* = 26 *LLS* individuals and *n* = 8 *HLS* individuals), 2014 (*n* = 106 *LLS* individuals and *n* = 20 *HLS* individuals), and 2015 (*n* = 75 *LLS* individuals and *n* = 16 *HLS* individuals). (*E*) *HKT1;1* mRNA expression in roots of T13^LLS^ and JBB^LLS^ plants (black bars), and T13^HLS^ and JBB^HLS^ plants (red bars) grown in coastal and inland common gardens. Data represent the mean ± SE (*n* = 3 plants per accession and field site).

If maladaptation provides a boundary for *HKT1;1*^*HLS-1*^ migration to sites very close to the sea, what maintains *HKT1;1*^*HLS-1*^ at the sites with intermediate soil salinity? Focusing on the six demes where we found that *HKT1;1*^*LLS*^ and *HKT1;1*^*HLS*^ cooccur, we observed that the number of plants with the *HKT1;1*^*HLS-1*^ allele within each deme varies depending on the year we sampled and that *HKT1;1*^*HLS-1*^ was generally the minor allele with the exception of one deme (T13) in which more than half of the plants harbored *HKT1;1*^*HLS-1*^ (Fig. 3*A*). There is no clear spatial pattern in the distribution of the two alleles within demes; plants containing *HKT1;1*^*HLS-1*^ were interspersed with plants containing *HKT1;1*^*LLS*^ throughout the demes (Fig. 3*A*). Strikingly, despite the exclusion of *HKT1;1*^*HLS-1*^ from the most extreme saline coastal demes, the frequency of *HKT1;1*^*HLS-1*^ in these six demes was found to correlate (*r*^2^ = 0.498) with the soil sodium concentration at the demes over the 3 y of our analysis (Fig. 3*B*), with years and sites with higher soil sodium favoring the *HKT1;1*^*HLS-1*^ allele. From these results we conclude that *HKT1;1*^*HLS-1*^ is favored in soil with an intermediate level of salinity (75–100 mg/g) (Fig. 2*A*), but beyond this limit it is maladaptive.

Consistent with this hypothesis that the *HKT1;1*^*HLS-1*^ allele is adaptive on moderately saline soils, we found a clear phenotypic effect of the *HKT1;1* allele on plant growth in moderate saline conditions. Growth of plants with the *HKT1;1*^*LLS*^ allele in soil was reduced after irrigation with 50 mM NaCl, while plants harboring the *HKT1;1*^*HLS-1*^ allele exhibited no growth inhibition with the same treatment (*SI Appendix*, Fig. S6*A*). Similar results were seen in growth experiments in hydroponics (*SI Appendix*, Fig. S6*B*). This robust growth effect was also borne out when observing silique number as a proxy for fitness of plants cultivated in soil. Plants harboring either the *HKT1;1*^*LLS*^ or the *HKT1;1*^*HLS-1*^ allele produced the same number of siliques in the absence of treatment with NaCl. However, after treatment with 50 mM NaCl silique number was reduced only in plants with the *HKT1;1*^*LLS*^ allele (Fig. 3*C*). This is fully consistent with the recent observations by An et al. (4) showing that *HKT1;1*^*HLS-1*^ is responsible for the elevated salinity tolerance previously observed in the Tsu-1 accession (3). These results support our conclusion that *HKT1;1*^*HLS-1*^ is adaptive to the intermediate soil salinity that occurs within the zone in which this allele is naturally confined in Catalonia.

As has been elegantly shown in ref. 4, the *HKT1;1*^*HLS-1*^ allele confers elevated salinity tolerance by limiting Na accumulation in reproductive tissues. This is achieved by enhanced *HKT1;1* expression specifically in stems, allowing Na to be retrieved from the xylem before transfer to the inflorescence. Interestingly, the polymorphisms in the promoter of *HKT1;1*^*HLS-1*^ that drive enhanced expression in the stem appear also to be responsible for the reduced expression in roots previously observed to drive elevated leaf Na (3, 6). This suggests the following trade-off: Enhanced salinity tolerance through increased stem expression of *HKT1;1* limits Na accumulation in reproductive tissues, while at the same time low root expression of *HKT1;1* allows overaccumulation of Na in leaves. We suggest that this trade-off provides a clue as to why *HKT1;1*^*HLS-1*^ is confined to a zone of intermediate soil salinity in its native habitat. Exclusion of Na from reproductive tissues provides enhanced tolerance to soil salinity only as long as the elevated leaf Na that is associated with it does not exceed a tolerable limit. This limit will be exceeded if plants containing *HKT1;1*^*HLS-1*^ migrate to the sites with the highest soil salinity closest to the coast.

To confirm that plants with *HKT1;1*^*HLS-1*^ from our Catalonian demes accumulate more Na^+^ in leaves than *HKT1;1*^*LLS*^-harboring plants, we incorporated genotypic data about the locus into a reanalysis of leaf Na data from (*i*) samples of each deme collected in the field in 2013, 2014, and 2015 (Fig. 3*D*); (*ii*) samples of each deme grown hydroponically under 0, 50, and 100 mM NaCl in 2014 and 2015 (*SI Appendix*, Fig. S6*C*); and (*iii*) samples from each deme cultivated in BLA and SCF common gardens in 2014 and 2015 (*SI Appendix*, Fig. S7). Leaf Na data from all these analyses confirmed that the presence of *HKT1;1*^*HLS-1*^ is associated with substantially increased Na^+^ concentration in leaves of plants grown the field and those grown experimentally (Fig. 3*D* and *SI Appendix*, Figs. S6*C* and S7). This elevated leaf Na in plants with *HKT1;1*^*HLS-1*^ is also associated with reduced expression of *HKT1;1* in the roots of plants grown in our common field gardens (Fig. 3*E*) and hydroponically (*SI Appendix*, Fig. S6*D*), as previously observed (3, 4, 6).

### Evidence for Fluctuating Selection in *HKT1;1* Mixed Demes.

If *HKT1;1*^*HLS-1*^ is favored in soils with salinity levels intermediate between the coast and inland, what maintains both *HKT1;1*^*LLS*^ and *HKT1;1*^*HLS-1*^ at these locations? There are two obvious possibilities: that fluctuating selection may maintain this polymorphism in the same deme or that *HKT1;1*^*LLS*^ migrates into this intermediate zone from either coastal or inland demes where *HKT1;1*^*LLS*^ is the predominant allele.

**Fig. 4.**
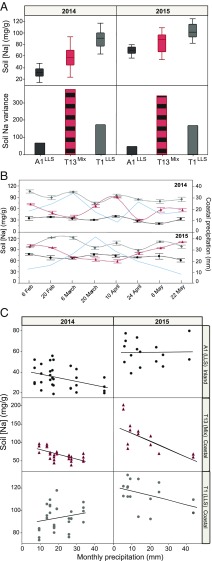
Soil sodium variability during the *A. thaliana* growing season. (*A* and *B*) Mean ± SE and variance in soil Na content (*A*) and soil Na concentration over time (*B*) of two independent samples from A1 (an inland site with all plants having the *HKT1;1*^*LLS*^; black), T13 (a coastal site containing a mix of plants harboring the *HKT1;1*^*LLS*^ allele or the *HKT1;1*^*HLS*^ allele; red/black bars), and T1 (a coastal site with all plants having the *HKT1;1*^*LLS*^ allele; gray bars) collected twice a month from February through May in 2014 and 2015. (*C*) Correlation between soil Na content and total monthly precipitation from January to June of 2014 and 2015 in the A1 [inland, *HKT1;1*^*LLS*^ allele (*LLS*), black dots], T13 [coastal, mixture of plants with the *HKT1;1*^*LLS*^ allele or the *HKT1;1*^*HLS*^ allele (Mix), red/black triangles], and T1 [coastal, *HKT1;1*^*LLS*^ allele (*LLS*), gray dots] sites.

All the current evidence supports the conclusion that *HKT1;1*^*HLS-1*^ provides a level of tolerance to moderately elevated soil salinity. Therefore, if fluctuating selection is responsible for maintaining both *LLS* and *HLS-1* alleles of *HKT1;1* at our mixed demes, then one possible agent of selection that might vary temporarily is soil salinity. To test this hypothesis, we selected a deme very close to the coast (T1, Tossa de Mar, located 100 m from the sea), a mixed deme containing both alleles of *HKT1;1* (T13, Tossa de Mar, located 530 m from the sea and less than 1 km from the T1 site), and a deme at an inland site (A1, Anglès, located 33 km inland from the coast). We collected soil samples twice a month from February through May of 2014 and 2015 to analyze the variability in soil Na during the *A. thaliana* growing season. We also investigated the relationship between soil Na and precipitation using data obtained from the weather stations of Tossa de Mar and Anglès. As expected, we observed that average soil Na is highest at our site closest to the sea (T1), is lowest inland (A1), and is intermediate at our site containing the deme with both *HKT1;1*^*HLS-1*^ and *HKT1;1*^*LLS*^ alleles (T13) (Fig. 4*A*). We would expect *HKT1;1*^*HLS-1*^ to be favored at this intermediate soil Na concentration at T13. However, in both years of our study the variation in soil Na concentration at the T13 site containing both *LLS* and *HLS* alleles was more than double that observed at either T1 or A1 (Fig. 4*A*). Moreover, this enhanced variability can lead to soil Na concentrations at T13 reaching those observed at T1, our site closest to the sea, as occurred in April 2014 and May 2015 (Fig. 4*B*). If soil Na concentrations suddenly increase to levels normally observed only at sites very close to the coast, as can happen at T13, the *HKT1;1*^*HLS-1*^ allele should be maladaptive (Fig. 2). Such fluctuations in soil Na would then maintain both the *LLS* and *HLS-1* alleles of *HKT1;1*.

By relating soil Na concentrations to rainfall at our three sites across both years, we observed that the increased variability in soil Na at T13 is coupled with variation in rainfall, with soil Na and rainfall being correlated at T13 (*r*^2^_2014_ = 0.427; *r*^2^_2015_ = 0.406) (Fig. 4*C*). Soil Na increases at T13 in months with low rainfall and decreases in months with high rainfall. However, no such relation was observed (*r*^2^ < 0.2) at our sites closest to the coast (T1) or inland (A1) (Fig. 4*C*).

It has been argued that salt spray is one of the main determinants of the makeup of coastal vegetation (31). The amount of deposition from salt spray is very much influenced by wind velocity, land topography, and rainfall, decreasing strongly within 600–800 m from the sea (32). We speculate that soil Na concentrations at our sites closest to the sea may be predominantly controlled by sea spray, whereas sea spray has less influence at our intermediate sites slightly back from the coast and more sheltered from onshore winds, such as T13 that contains both *LLS* and *HLS-1* alleles of *HKT1;1*, allowing rainfall to become the predominate factor controlling soil Na (33).

### *HKT1* Origin and Migration.

**Fig. 5.**
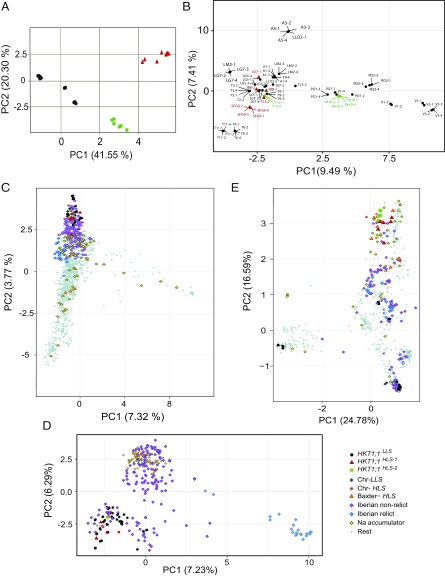
Population genomic relationships of 1,212 *HKT1;1* alleles and their harboring genomes. (*A*) PCA of the *HKT1;1* locus region (12 kb) of 74 *A. thaliana* individuals from 19 demes. Black dots, *HKT1;1*^*LLS*^; red triangles, *HKT1;1*^*HLS-1*^; green squares, *HKT1;1*^*HLS-2*^. (*B*) Genome-wide PCA of 77 *A. thaliana* genomes from 19 demes. Black, *HKT1;1*^*LLS*^; red, *HKT1;1*^*HLS-1*^; green, *HKT1;1*^*HLS-2*^. (*C*) PCA of genome-wide polymorphism data. (*D*) PCA of genome-wide polymorphism data excluding the noninformative 891 worldwide *A. thaliana* accessions. (*E*) PCA of *HKT1;1* locus data of all 77 Catalonian and 1,135 *A. thaliana* genomes (1001genomes.org/). Black dots, 60 *HKT1;1*^*LLS*^ individuals; red triangles, eight *HKT1;1*^*HLS-1*^ individuals; green squares, six *HKT1;1*^*HLS-2*^ individuals; black asterisk, two *HKT1;1*^*LLS*^ de novo individuals; red asterisk, one *HKT1;1*^*HLS-1*^ *de novo* individual; orange triangles, seven *HKT1;1*^*HLS*^ individuals (6); purple rhombus, 155 Iberian nonrelicts; blue rhombus, 22 Iberian relicts; yellow rhombus, 60 worldwide Na accumulators; gray dots, 891 worldwide *A. thaliana* accessions.

To gain an understanding of the demographic history and population genomic relationships between plants harboring each of the three *HKT1;1* alleles identified in our study site, we performed a large-scale genomic analysis. First, principle component analysis (PCA) of the *HKT1;1* region (promoter + gene) supports the three *HKT1;1* groups we previously identified (Fig. 5*A*). These three groups are consistent with our allelic paradigms, *HKT1;1*^*HLS-1*^ (red triangles), *HKT1;1*^*LLS*^ (black circles), and *HKT1;1*^*HLS-2*^ (green squares).

Moving from the *HKT1;1* locus to whole-genome population comparisons within our Catalonian study site, we see completely different relationships. In contrast to the clear grouping of the three *HKT1;1* alleles, whole-genome PCA of the same individuals shows that genomes harboring each allele type are highly interspersed (Fig. 5*B*), with genomic variation outside the *HKT1;1* locus dominating groupings. However, in the two mixed demes included in the whole-genome sequencing (LG7 and T13), individuals with *HKT1;1*^*HLS-1*^ or *HKT1;1*^*HLS-2*^ do not cluster closely with the other individuals from the same deme, suggesting that individuals with the *HKT1;1*^*HLS*^ alleles have a different origin from the individuals with the *HKT1;1*^*LLS*^ allele. We also see evidence of local migration events between demes. In LLO2, which shows high clonality of three individuals, we see one individual, LLO2-1, that genetically clusters with the individuals from the A5 deme, suggesting that seeds from A5 (inland) plants migrated to the LLO2 (coastal) deme although they are 40 km apart. The other case suggests a 10-km migration event with LM2-1 clustering with individuals from the LG7 deme. Overall, however, despite the limited migration we detect, most of the demes are highly clonal. Indeed, we count only on average ∼50–70 private alleles on each chromosome between demes. Thus, the majority of the demes are locally isolated, although cases of migration exist, mostly from inland to the coast and along roads.

To gain a worldwide picture of the distribution of the *HKT1;1* alleles we identified, and to gain insight to their demographic histories, we contextualized our 77 Catalonian genomes by framing them with the 1,135 *A. thaliana* genomes available at 1001genomes.org/, merging them into a single dataset of 1,212 sequenced genomes (Fig. 5 *C*–*E*). Leaf ionomic profiles of the 1,135 *A. thaliana* accessions with sequenced genomes (Dataset S2) were used to guide the identification of 60 accessions we term here as “Na accumulators.” Under control conditions these accessions accumulated more than 1,750 μg/g of Na in leaves (the 5% tail of the empirical distribution). Surprisingly, at the genome level, all the Catalonian samples are relatively clustered with the Iberian nonrelicts and the bulk of the European accessions (Fig. 5 *C* and *D*). That is, *HKT1;1*^*LLS*^, *HKT1;1*^*HLS-1*^^*&*^^*HLS-2*^-harboring plants from our study are not clustered with the Iberian relicts that have remained in Catalonia since before the last glaciation. This contrasts interestingly with work suggesting candidate barrier loci in the Iberian accessions (34).

However, focusing again on the *HKT1;1* locus, a different picture emerges (Fig. 5*E*). We see strong separation between *HKT1;1* allele types, with the adaptive high leaf Na accessions clustering more closely with the broadly distributed high leaf Na allele described in ref. 6 at the top of the principle component 2 axis. These saline-adapted alleles also cluster relatively closely to some Iberian nonrelicts rather than to any Catalonian *HKT1;1*^*LLS*^ -harboring samples or to Iberian relicts. Given the lack of clustering of the *HKT1;1*^*HLS-1*^^*&*^^*HLS-2*^ allele-containing plants with the Iberian relicts, we conclude that the adaptive *HKT1;1* alleles are not native Iberian relicts but instead are more recent migrants to the region. This supports the hypothesis of the recent expansion of a “cosmopolitan” *A. thaliana* and that *A. thaliana* is the product of a dramatic series of range expansions (34), upon which the adaptive *HKT1;1* allele rode into Catalonia as a relatively recent tourist. Finally, we observe evidence confirming that variation at the *HKT1;1* locus is a worldwide determinant of Na accumulation: both *HKT1;1*^*HLS-1*^ and *HKT1;1*^*HLS-2*^ individuals cluster together and with the majority of worldwide Na accumulators (Fig. 5*E*, yellow rhombuses relatively enriched in the top right cluster). This strongly suggests that many of these Na accumulators contain *HLS-1* or *HLS-2* alleles of *HKT1;1* that have not yet been identified.

## Conclusion

While soil salinity is a critical factor limiting plant growth and productivity worldwide, there has been a lack of understanding of the factors that mediate saline adaptation dynamically over time and space. Fewer cases have elucidated the complete thread through the population genomic, ecological, and mechanistic bases of saline adaptation. In this study, we have bridged these gaps. Further, we have confirmed at a genomic level that variation at the *HKT1;1* locus is a major worldwide determinant of saline adaptation and suggest that rapidly varying within-deme dynamics of the *HKT1;1* alleles allows rapid adaptation to highly volatile fluctuating seasonal soil-salinity dynamics.

## Materials and Methods

Detailed descriptions of samples and methods are provided in *SI Appendix*. All sequence data are freely available in the National Center for Biotechnology Institute Sequence Read Archive database (BioProject PRJNA506760).

### Collection of Plant and Soil Material.

Thirty-six demes of *A. thaliana* were selected from the northeast of Catalonia. A “deme” is defined as a small group or stand of *A. thaliana* plants growing in relatively homogeneous ecological conditions and separated from other groups by at least 35 m. Since 2013–2015, samples were collected annually from each site; one-half of the plant was used for ionomic analysis, and the other half was used to extract DNA for *HKT1;1* genotyping. Seeds of each individual were collected directly in the field and stored in packets over silica gel in a sealed box.

For soil elemental analysis, we collected three soil samples (∼50 g of soil from the top 10 cm of soil) at each site during the first week of May of 2013, 2014, and 2015. Twenty grams of soil was kept intact at 4 °C to analyze the physical properties, and the rest was air-dried under laboratory conditions, passed through a 2-mm sieve, and stored in a dry place. To monitor soil properties, three demes (T1, T13, and A1) were selected, and soil samples were collected twice a month (from February to June).

We obtained monthly precipitation data for T13, T1, and A1 demes from the Tossa de Mar and Anglès weather stations (www.meteo.cat).

### SNP Genotyping and Expression (qRT-PCR) of *HKT1;1*.

DNA and RNA were extracted from frozen tissue following the methods described in *SI Appendix*, section S1. A derived cleaved amplified polymorphic sequence (dCAPS) marker was developed based on the C/T SNP on *HKT1;1* at Chr4:6392280 with forward primer 5′-AAGAGACGGTGATGGCAGAG-3′ and reverse primer 5′-GAGGGCGAAATCTTCACCTCCT-3′. A 2-bp mismatch was made in the reverse primer, generating a *XhoI* site in the *HKT1;1*^*LLS*^ (CT**C**GAG) but not in the *HKT1;1*^*HLS-1*^ (CT**T**GAG).

Root expression of *HKT1;1* was calculated with the 2^−ΔCt^ method (35), using *PP2A* gene (At2g37620) and *HKT1;1* (At4g10310) transcript quantification. For each sample, the average value from triplicate real-time PCRs was used to estimate transcript abundance. Primers and method are detailed in *SI Appendix*, section S1.

### Field-Based Reciprocal Common Garden Experiments.

After genotyping plants from the 2012 and 2013 collections, we selected two demes containing plants with the *HKT1;1*^*HLS-1*^ and the *HKT1;1*^*LLS*^ alleles; we named these demes “T13” and “JBB.” In March 2014 and 2015, 100 seeds of each deme/*HKT1;1* variant (T13^*LLS*^, T13^*HLS-1*^, JBB^*LLS*^, and JBB^*HLS-1*^) were sown into 16 × 16 cm squares at the BLA coastal field (N 41°40′37.64″; E 2°48′3.86″) and at the SCF inland field (N 41°50′41.04″; E 2°40′36.13″). Two weeks after germination, we left two plants in each square. We studied the fitness of 10 plants for each deme/*HKT1;1* variant at each site. The other 10 plants were harvested in April 2014 and 2015 to analyze leaf ionome and *HKT1;1* root expression. Rosette diameter was measured every week for 2 mo, and the number of siliques was counted at maturity. To measure flowering time, we recorded the date when the first flower of each plant appeared.

### Salinity Tolerance Assays.

The same four demes selected for the common field garden experiment were used to perform irrigation and hydroponics experiments (*SI Appendix*, section S2) with 0, 50, and 100 mM NaCl to test salinity tolerance, analyze leaf ionome, and quantify expression of *HKT1;1* in roots. To examine the fitness effects of elevated salinity, we measured rosette diameter every 3–4 d and counted the number of siliques produced at maturation. Leaves and roots were weighed at the time of harvest. To test for differential accumulation of Na in aerial tissue, two leaves of each plant were harvested, dried at 60 °C for 2 d, and stored for inductively coupled plasma (ICP)-MS analysis. Roots of each plant were put in Eppendorf tubes, frozen with liquid nitrogen immediately after being cut, and stored in the freezer at −80 °C for subsequent RNA extraction.

### Soil and Leaf Elemental Analysis.

Five grams of soil was dried for 42 h at 60 °C in 50-mL Falcon tubes. The extraction method (adapted from ref. 36) is detailed in *SI Appendix*, section S3. Each sample was diluted to 6.0 mL with 18 MΩ water and analyzed for As, B, Ca, Cd, Co, Cu, Fe, K, Li, Mg, Mn, Mo, Na, Ni, P, Rb, S, Se, Sr, and Zn on an ELAN-DRCe ICP-MS instrument (PerkinElmer Sciex). National Institute of Standards and Technology (NIST) traceable calibration standards (ULTRA Scientific) were used for calibration.

Plants from the field or laboratory were sampled by removing two or three leaves (1–5 mg dry weight), which were washed with 18 MΩ water before being placed in Pyrex digestion tubes. Sampled plant material was dried for 42 h at 60 °C and weighed before open-air digestion in Pyrex tubes using 0.7 mL concentrated HNO_3_ (Mallinckrodt AR select grade) at 110 °C for 5 h. Each sample was diluted to 6.0 mL with 18 MΩ water and analyzed for As, B, Ca, Cd, Co, Cu, Fe, K, Li, Mg, Mn, Mo, Na, Ni, P, Rb, S, Se, Sr, and Zn on an ELAN-DRCe ICP-MS instrument (PerkinElmer Sciex). NIST traceable calibration standards (ULTRA Scientific) were used for calibration.

### Sequencing, Data Processing, and Genome Assembly.

Leaf material from 74 individuals from 19 *A. thaliana* demes (four individuals from each deme except for two individuals from JBB) (Dataset S1) were used for DNA extraction (*SI Appendix*, section S4) and genome resequencing. Genome resequencing was performed on an Illumina HiSeq 2500 sequencing system in paired-end mode with data processing described in *SI Appendix*, section S5.

Frozen leaf material from one individual of the JBB, T11, and PA10 demes was used to perform de novo genome assemblies with linked reads generated with the 10× Genomics Chromium platform and Supernova assembler (*SI Appendix*, section S6). Samples S1 (T11), S2 (JBB), and S12 (PA10) were sequenced on an Illumina HiSeq 2500 sequencing system in rapid run mode (250-bp or 150-bp paired-end sequences). Fragment sizes were assessed using a Q-card (OpGen Argus optical mapping system) and the Genomic DNA TapeStation assay (*SI Appendix*, Table S5); assembly metrics are described in *SI Appendix*, Table S6.

### Alignment, PCA, and Phylogenetic Analysis.

We visualized, aligned, and blasted sequences obtained from the assemblies to National Center for Biotechnological Information database with Geneious 10.0.9 software (37). HybridCheck (38) was used to investigate the similarity of the three *HKT1;1* alleles.

PCAs were performed both on the *HKT1;1* locus and on the genome-wide SNP data using the glPCA function of the adegenet R package (39). PCAs were visualized using ggplot2 (40). The genome-wide SNP dataset was linkage disequilibrium-pruned using custom scripts and further filtered to include only putatively neutral fourfold degenerate sites. PCAs were performed for both SNP datasets using the 77 described samples as well as worldwide *A. thaliana* accessions from the 1,135 *A. thaliana* genomes dataset (1001genomes.org/). The worldwide *A. thaliana* accessions were classified in five categories: *HKT1;1* weak accessions (*n* = 7) described in ref. 6; Iberian nonrelicts (*n* = 155); Iberian relicts (*n* = 22) described in ref. 23; accessions that accumulate more than 1,750 μg/g of Na in leaves under control conditions (*n* = 60) (Dataset S2); and the rest of the worldwide *A. thaliana* accessions (*n* = 891).

To obtain the *HKT1;1* cladogram, we generated a progressive alignment of 138 SNPs from 79 plants [including Col-0 and the African relict Atlas (41)]. Pairwise genetic distance between individuals and between demes was calculated using the maximum likelihood statistical method and the Jukes and Cantor substitution model. The cladogram was rooted by the African *A. thaliana* relict individual (41). STRUCTURE 2.3.3 (42) was used to visualize the three versions of *HKT1;1* in a structure plot with three clusters (K = 3). The number of genetic clusters (K) was set from 1 to 10, and 10 runs were performed for each genetic cluster with 1 million Markov chain Monte Carlo (MCMC) iterations using the admixture ancestry model with correlated allele frequencies.

### GWA Analysis.

Genotype data of the 1,135 *A. thaliana* accessions were obtained from 1001genomes.org/ (1135_snp-short-indel.vcf.gz). Indels were removed, and SNPs were filtered using the criteria allele count <100 and minor allele frequency <4.8% (the frequency of our triallelic *HKT1;1* site at Chr4:6392067) using GATK-3.6.0 (43), leaving a total of 1,590,315 SNPs. Phenotype data—normalized leaf Na values for the 1,135 strains—can be found in Dataset S2. Procedures and software used for GWAS analysis are described in *SI Appendix*, section S7. Bonferroni correction was adopted for multiple tests of all genes in the genome. A gene was considered significant at the genome-wide significance level if the nominal *P* value was less than 0.01/N. GWAS outputs can be found in *SI Appendix*, Fig. S4.

### Protein Modeling.

A homology model of dimeric *A. thaliana* *HKT1;1* was generated with Modeler 9.19 (44) using the structures of *Vibrio parahaemolyticus* TrkH [Protein Data Bank (PDB) ID code 3PJZ] (45) and *Bacillus subtilis* KtrAB (PDB ID code 4J7C) (46) as templates. The final model was determined by its discrete optimized protein energy (DOPE) score. Multiple sequence alignments were generated using Clustal Omega (47) with sequences from Phytozome (48) and GenBank (49). Colors of *SI Appendix*, Fig. S2 were generated using Jalview 2.9 (50).

### Statistical Analysis.

One-way ANOVA was used to test for significant differences between means of fitness, elemental contents of soil, leaf material, and gene expression. Correlations between two variables used Bivariate fit. Multiple comparisons of group means used Tukey’s honestly significant difference (HSD) and Dunnett’s tests. All analyses were performed in JMP 13.0 (51).

## Supplementary Material

Supplementary File

Supplementary File

Supplementary File
