# Supplementary material for: Fluctuating selection on migrant adaptive sodium transporter alleles in coastal Arabidopsis thaliana
Source: Proc Natl Acad Sci U S A. 2018 Dec 7;115(52):E12443–52. doi: 10.1073/pnas.1816964115 (PMC6310793; doi:10.1073/pnas.1816964115)
Supplement: Supplementary File [file pnas.1816964115.sapp.pdf]

PNAS
www.pnas.org

Supplementary Information for

Fluctuating selection on migrant adaptive sodium transporter alleles in coastal *Arabidopsis thaliana*

Silvia Busoms[1,2], Pirita Paajanen[1], Sarah Marburger[1], Sian Bray[1], Xin-Yuan Huang[3], Charlotte Poschenrieder[2], Levi Yant[1,4], and David E Salt[5]

1. Department of Cell and Developmental Biology, John Innes Centre, Norwich Research Park, Norwich, NR4 7UH, United Kingdom
2. Department of Plant Physiology, Universitat Autònoma de Barcelona, Spain
3. State Key Laboratory of Crop Genetics and Germplasm Enhancement, College of Resources and Environmental Sciences, Nanjing Agricultural University, Nanjing 210095, China
4. School of Life Sciences, University of Nottingham, UK
5. Division of Plant and Crop Sciences, School of Biosciences, University of Nottingham, UK

Corresponding Author: Levi Yant (Levi.Yant@nottingham.ac.uk) and David E Salt (david.salt@nottingham.ac.uk)

This PDF file includes:

Supplementary text S1 to S7
Figs. S1 to S7
Tables S1 to S5

Other supplementary materials for this manuscript include the following:

Datasets S1 and S2

Supplementary Information Text

Section S1. DNA and RNA extractions for *HKT1;1* SNP genotyping and root expression

DNA was extracted from frozen leaf tissue using 50 mM TRIS (pH 9) and 5 mM EDTA (pH 8). After heating at 95°C for 5 min, 4µl of extract was directly used as a template for PCR. 10 µl PCR reactions contained 2µl 5X Green GoTaq® reaction buffer (Promega), 0.8 µl 25mM $MgCl_2$, 0.8 µl 2.5 mM dNTPs, 0.4 µl 10 mM forward and reverse primer and 0.3 µl home-made Taq polymerase. A total 45 cycles PCR was performed with 30 sec at 94°C, 15 sec annealing at 60°C followed by 30 sec extension at 72°C. PCR product was then digested with *XhoI* overnight and separated on 3% agarose gel.

Total RNA was extracted using the Qiagen RNeasy Plant Mini Kit (http://www.qiagen.com), and DNase digestion was performed during the extraction procedure according to the manufacturer's instructions. Two micrograms of total RNA were used as a template to synthesize first-strand cDNA with random hexamers using SuperScript II Reverse Transcriptase (Invitrogen Life Technologies, http://www.invitrogen.com). Quantitative real-time PCR was performed with the first strand cDNA as a template on a sequence detector system (ABI Prism 7000, Applied Bio-systems) with Maxima SYBR Green qPCR Master Mixes (Thermo Scientific).

For normalization across samples, the expression of the *PP2A* gene (At2g37620) was used with the following primers: *PP2A-F*, 5'-TAACGTGGCCAAAATGATGC-3' and *PP2A-R*, 5'-GTTCTCCACAACCGCTTGGT-3'. For *HKT1;1* (At4g10310) transcript quantification the following primers were used: *HKT-RTF*, 5'-TGG GATCTTATAATTCGGACAGTT C-3' and *HKT-RTR*, 5'-GATAAGACCCTCGCGATAATCAGT-3'. For each sample, the average value from triplicate real-time PCRs was used to estimate transcript abundance. Data was analysed using the SDS software (Applied Bio-systems version 1,0). Ct values were determined based on efficiency of amplification. The mean Ct values were normalized against PP2A gene and Ct values calculated as (Ct_{HKT1}- Ct_{PP2A}). The expression of *HKT1;1* was calculated with the $2^{-\Delta Ct}$ method.

Section S2. Irrigation and hydroponic experiments procedures

For the irrigation experiment, 15 plants of each deme/*HKT1;1* variant ($T13^{LLS}$, $T13^{HLS-1}$, JBB^{LLS} and JBB^{HLS-1}) were cultivated individually in square pots of 5 x 5 x 10 cm with potting mix soil. Seeds

were sowed on wet soil and the pots covered with PVC film until the seedlings had germinated. Pots with germinated seedlings were placed in a growth chamber (Conviron CMP5090) with 8 h light / 16 h dark photoperiod, an irradiance of 80 $mmol \cdot m^{-2} \cdot s^{-1}$ and a constant temperature of 22 ºC. Plants were watered with 0.5-strength Hoagland solution every 2-3 days. After 2 weeks 5 plants of each accession were irrigated once a week with 0.5-strength Hoagland solution containing 0, 50 or 100 mM NaCl. After two weeks of treatment the photoperiod was increased 2 h every 3 days until it reached 16 h light / 8 h dark to induce flowering. Rosette diameter was measured every 3-4 days during 3 weeks and the number of siliques produced was counted at maturation.

For hydroponics experiments, plants of each deme/*HKT1;1* variant were sown in 1,5 mL eppendorf tubes filled with vermiculite and distilled water and placed in a growth chamber (same conditions as previously). After emergence of the cotyledons the bottom 0,5 - 0,7 cm of each tube was removed to allow roots to grow into 0.5-strength Hoagland solution. When roots of the seedling were 2-3 cm long and the rosette diameter of the seedling was approximately 1,5 cm, 60 plants (20 per treatment) of $T13^{LLS}$, $T13^{HLS\text{-}1}$, JBB^{LLS} and $JBB^{HLS\text{-}1}$ were transferred to individually hydroponic circular containers (50 mL) filled with 0,5-strength Hoagland solution (pH 6,0). The hydroponic solution was changed every third day to maintain a consistent concentration of nutrients in the solution. Salinity treatment was initiated 14 days after transplantation by the addition of 0, 50 or 100 mM NaCl to the solution.

Section S3. Extraction methods for soil ionome analysis

Extraction method for soil samples consisted of a digestion with 20 mL of 1 M NH_4HCO_3, 0.005 M diaminetriaminepentaacetic acid, and 5 mL of pure water during 1 h of shaking on the rotary shaker at low speed. Each sample was gravity filtered through qualitative filter papers until obtaining approximately 5 mL of filtrate, which was transferred into Pyrex tubes; 0.7 mM trace grade c. HNO_3 was added and digested at 115°C for 4.5 h.

Section S4. Modified high molecular weight CTAP protocol for DNA extraction

A. thaliana leaf material (0.4 g) was ground in liquid nitrogen and 10ml of CTAB DNA extraction buffer was added (Tris-HCl 100mM, CTAB 2% (w/v), NaCl 1.4M, EDTA 20mM) and 20µl of Proteinase K at 20mg/ml. The mixture was incubated at 55°C for 1 hour, then cooled on ice. 0.5X volume Chloroform (Fisher Scientific) was added and inverted to mix. Samples were spun down at 3000rpm for 30 minutes. The upper phase was taken, and 1X volume of phenol:chloroform:isoamyl alcohol (25:24:1) was added and spun for 30 minutes at 3000rpm.

Again, to the upper phase only, 10% volume NaOAc at 3M was added along with 2.5X volume of ice cold 100% ethanol. The tubes were inverted to mix and incubated on ice for 30 minutes. The mixture was spun down for 30 minutes at 3000rpm at 4°C. The pellet was washed in 4ml of ice cold 70% ethanol (Sigma-Aldrich). Tubes were spun for 10 minutes at 3000rpm at 4°C. The 70% ethanol wash was repeated twice more. The pellet was air dried and resuspended in 300ul nuclease-free water with 3ul RNase A at 4mg/ml. DNA concentration was checked on a QuBit Flourometer 2.0 using the QuBit dsDNA HS Assay kit.

Section S5. Data processing for whole-genome resequencing

Genome resequencing of 74 *A. thaliana* individuals was performed on Illumina HiSeq 2500 in paired. Following demultiplexing and removal of adaptor sequences using 'Cutadapt' standard quality trimming was performed 'Trimmomatic' (settings: LEADING:10 TRAILING:10 SLIDINGWINDOW: 4:15 MINLEN:50) [1, 2]. Next, sequence data was processed to: (1) remove duplicate reads using Picard (MarkDuplicates); (2) apply a 'namefix' to the bam files using Picard (AddOrReplaceReadGroups) and (3) realign Indels using the GATK 'GenomeAnalysis' Toolkit [3]. Biallelic SNPs were identified using 'HaplotypeCaller' and genotyped using 'GenotypeGVCF' (both in GATK). Data was quality filtered using GATK SelectVariants using these parameters: QD < 2.0 || MQ < 40.00 || FS > 60.0 || SOR > 4.0 || MQRankSum < -8.0 || ReadPosRankSum < -8.0 and a minimum coverage of 10x per sample.

Section S6. 10X library construction and Supernova genome assembly

DNA from samples S1 (T11), S2 (JBB) and S12 (PA10) was diluted to 0.5 ng/μl with EB and checked with a QuBit Flourometer 2.0 using the QuBit dsDNA HS Assay kit. The Chromium User Guide was followed as per the manufacturer's instructions (10X Genomics, CG00043, Rev A). The final library was quantified using qPCR (KAPA Library Quant kit, ABI Prism qPCR Mix, Kapa Biosystems). Sizing of library fragments was confirmed using a Bioanalyzer (High Sensitivity kit, Agilent). Samples were pooled based on molarities calculated using the two QC measurements.

For the S1 (T11) sample 87.39 M were obtained and assembled with Supernova 1.1.4 (10x Genomics), giving effective coverage of 36.75x. The assembly size was 60 Mb (taking into account only contigs larger than 10 kb). Despite the fragmented assembly, we could identify the 25 kb contig that contained the *HKT1;1* locus. This was then confirmed and polished manually. For the S2 (JBB) sample 90.62 M reads were obtained and assembled with Supernova 1.1.4, giving

effective coverage of 63.69x. The assembly size was 100 Mb. The *HKT1;1*-locus was identified by BLAST and the relevant contig fetched by samtools faidx. For the S12 (PA10) sample we obtained 50.93 M reads, which we assembled with Supernova 1.1.4, giving an effective coverage of 64x and an assembly size of 110Mb. The *HKT1;1* locus was fetched again using samtools faidx.

The S1 and S12 Chromium samples were representative of $HKT1;1^{LLS}$ allele and S2 was representative of $HKT1;1^{HLS\text{-}1}$. Therefore, we necessary to assemble separately a sample from the PA10 population that harboured the $HKT1;1^{HLS\text{-}2}$. This was possible because the four resequenced PA10 plants were essentially clonal: we therefore pooled them together for sufficient coverage. We then used a hand-curated reference guided assembly method to create the third $HKT1;1^{HLS\text{-}2}$ locus.

Section S7. GWAS analysis

GWAS analysis was performed in two steps: first, a principle components analysis (PCA) was performed and a kinship matrix was calculated using the GAPIT package in R [4]. The "Q" matrix was determined by the PCA to account for effects due to population structure, and the kinship matrix (K) was calculated using the VanRaden algorithm [5] and the EMMA method to determine the familial relatedness. Secondly, the compressed mixed linear model (CMLM) was used for performing GWAS by incorporating K matrix along with PCAs employing the program TASSEL 5.0 [6].

Supplementary Figures

Figure S1

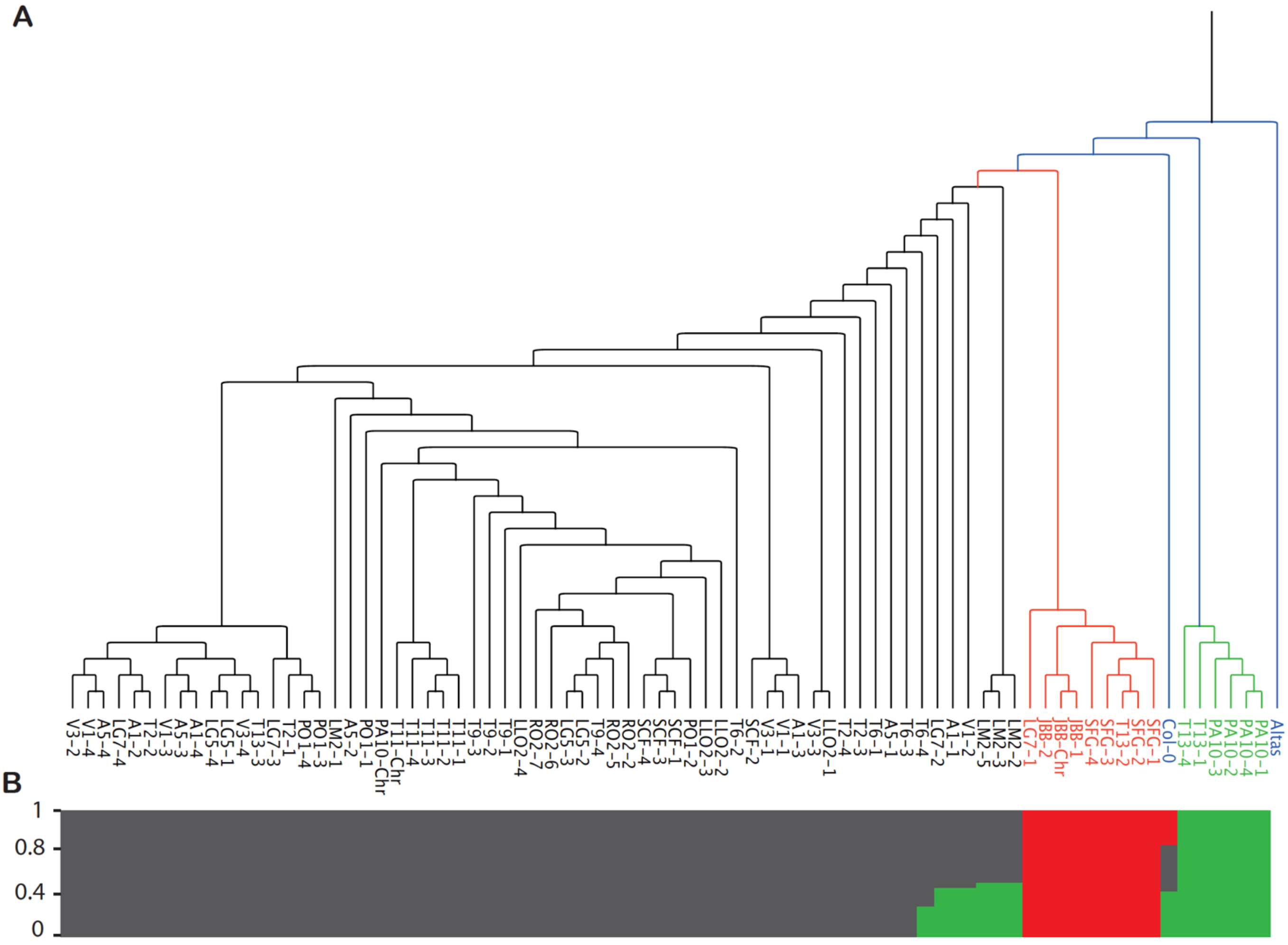

Fig. S1: (A) Neighbour-joining cladogram of *HKT1;1* allele (138 SNPs) among 77 Catalonian *A. thaliana* individuals, Col-0 reference and an African relict (Atlas). **(B)** Estimation of *HKT1;1* genetic structure within the 78 *A. thaliana* individuals. Each vertical bar represents an individual plant and each bar is divided into *K* coloured sections. Highest probability for the value of *K=3* classifies the three versions of *HKT1;1*: *HKT1;1*^{LLS} (black), *HKT1;1*$^{HLS-1}$ (red), *HKT1;1*$^{HLS-2}$ (green).

Figure S2

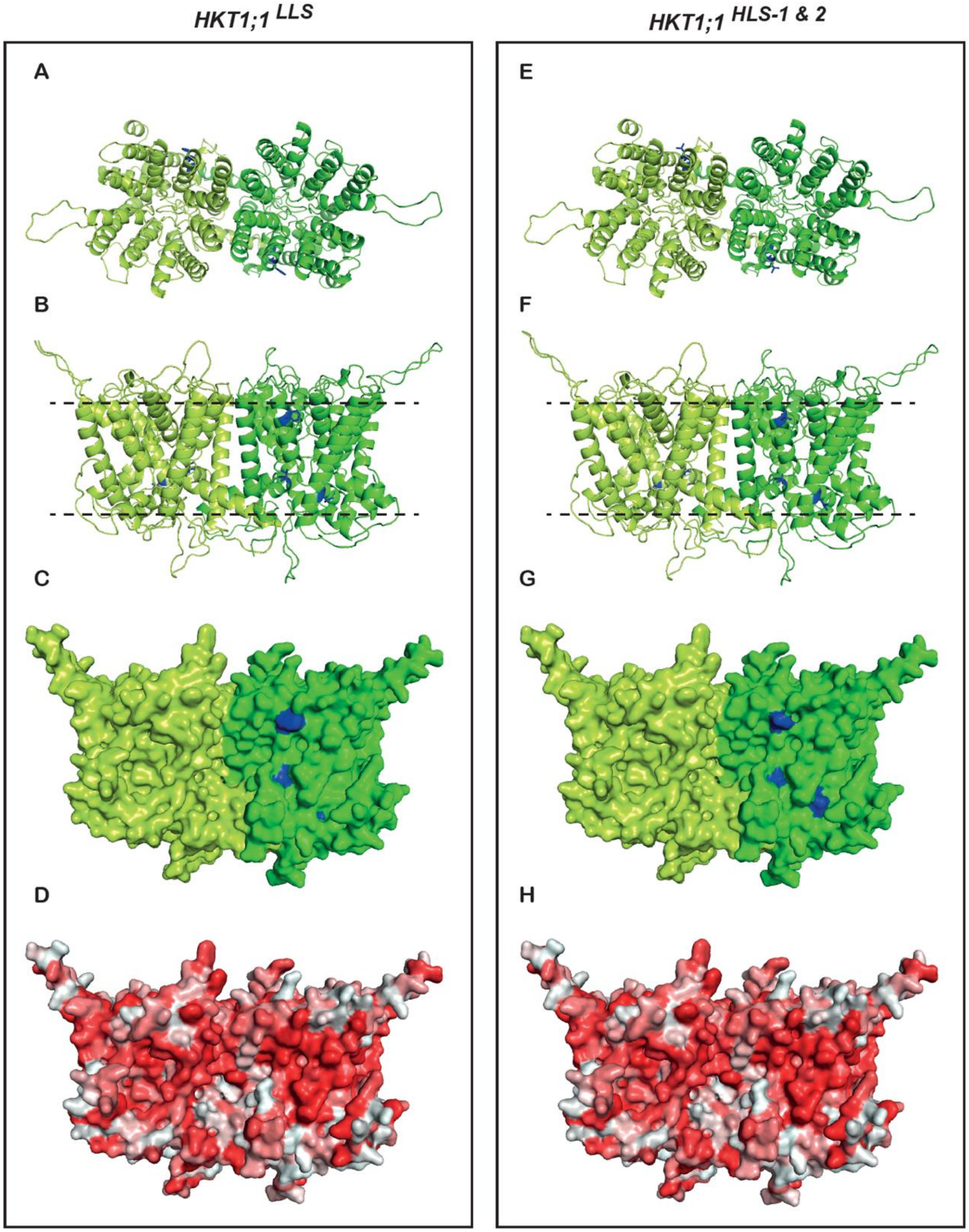

Fig. S2: Homology models of *Arabidopsis thaliana HKT1;1* dimer. The two monomers of the dimer are in shades of green. Fixed differences between *HKT1;1*$^{HLS\text{-}1\ \&\ 2}$ and *HKT1;1*^{LLS} alleles shown in blue. **(A-D)** *HKT1;1*^{LLS} allele. **(E-H)** *HKT1;1*^{HLS} alleles. **(A & E)** Top view from the outside of the cell. **(B-D & F-H)** Side view of the dimer; dotted line indicated the expected position of the membrane. **(D & H)** Hydrophobic nature of the protein surface plotted red (most hydrophobic) to white (least hydrophobic).

Figure S3

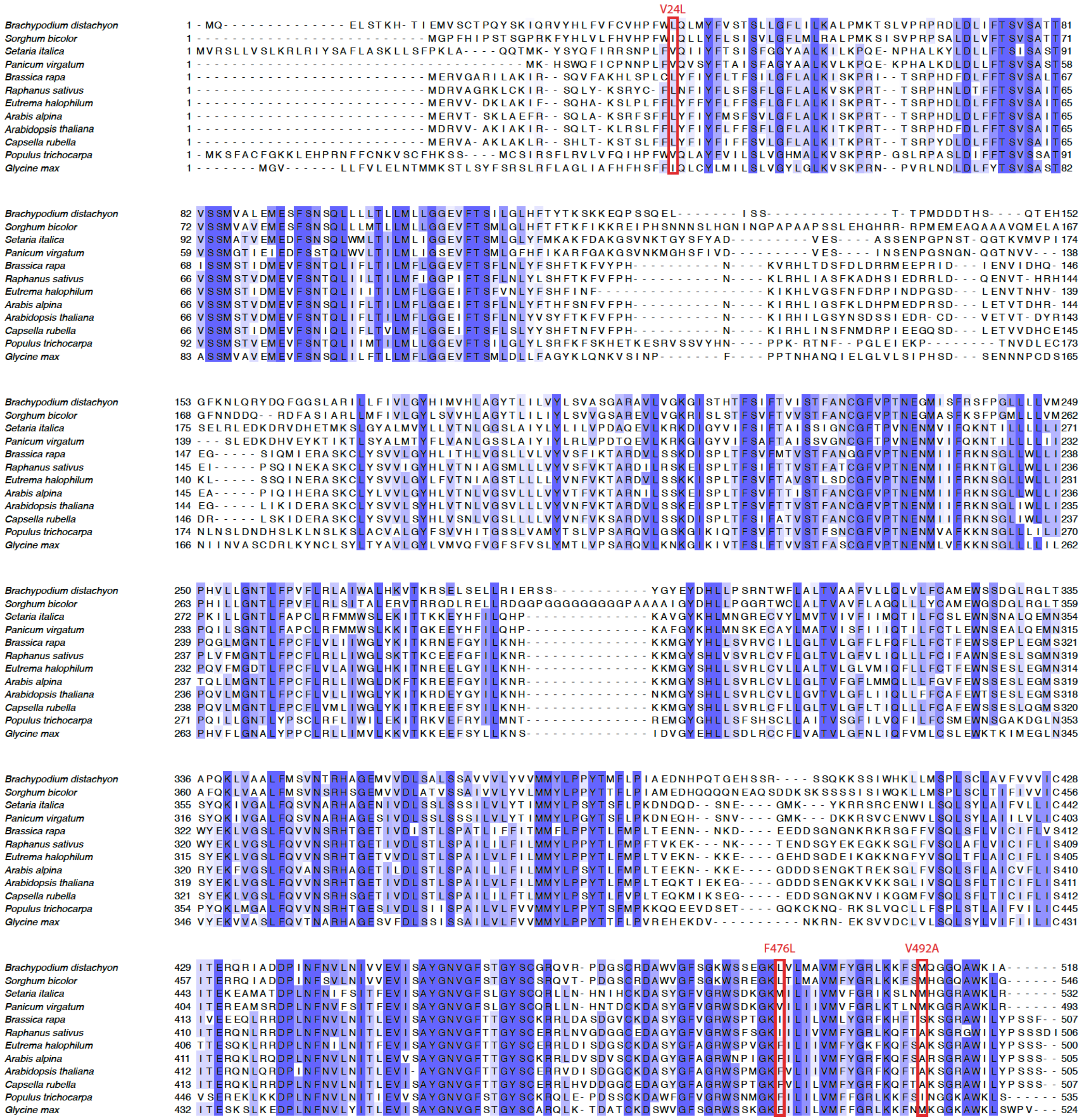

Fig. S3: Poor sequence conservation of the fixed amino acid differences between *HKT1;1*^{LLS} and *HKT1;1*$^{HLS\text{-}1\ \&\ 2}$ alleles. Multiple sequence alignment of *HKT1;1* sequences (*Brachypodium distachyon, Sorghum bicolor, Setaria italica, Panicum virgatum, Brassica rapa, Raphanus sativus, Eutrema halophilum, Arabis alpina, Arabidopsis thaliana, Capsella rubella, Populus trichocarpa, Glycine max*). Residues are coloured according to the percentage that match the consensus sequence from 100% (dark blue) to 0% (white). The positions of fixed differences between the alleles are highlighted with red rectangles.

Figure S4

(A) Whole-genome Manhattan Plot:

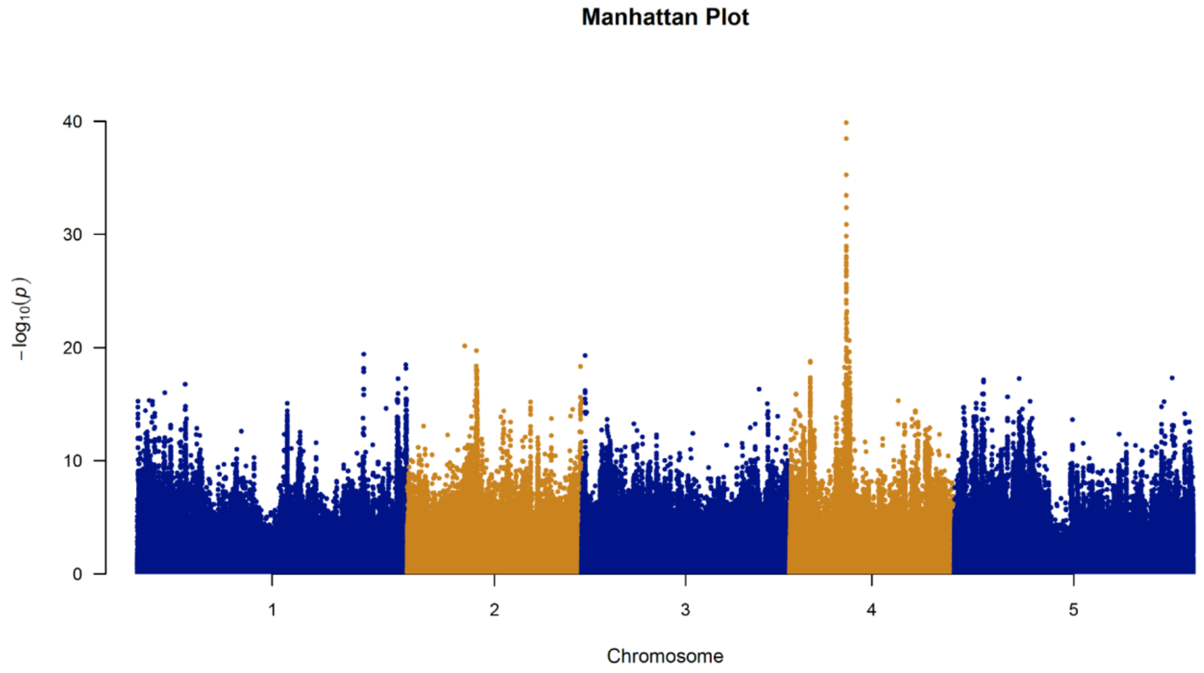

(B) Whole-genome Q-q plot:

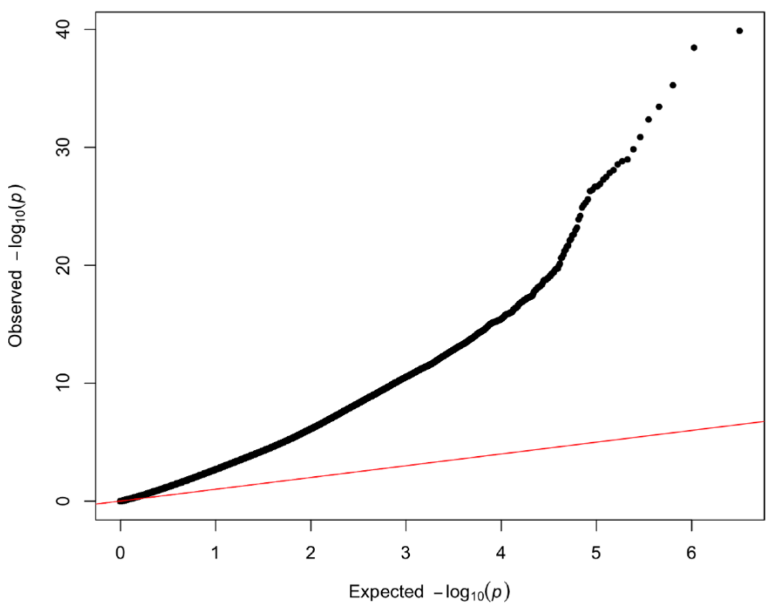

(C) Chromosome 4 Manhattan Plot:

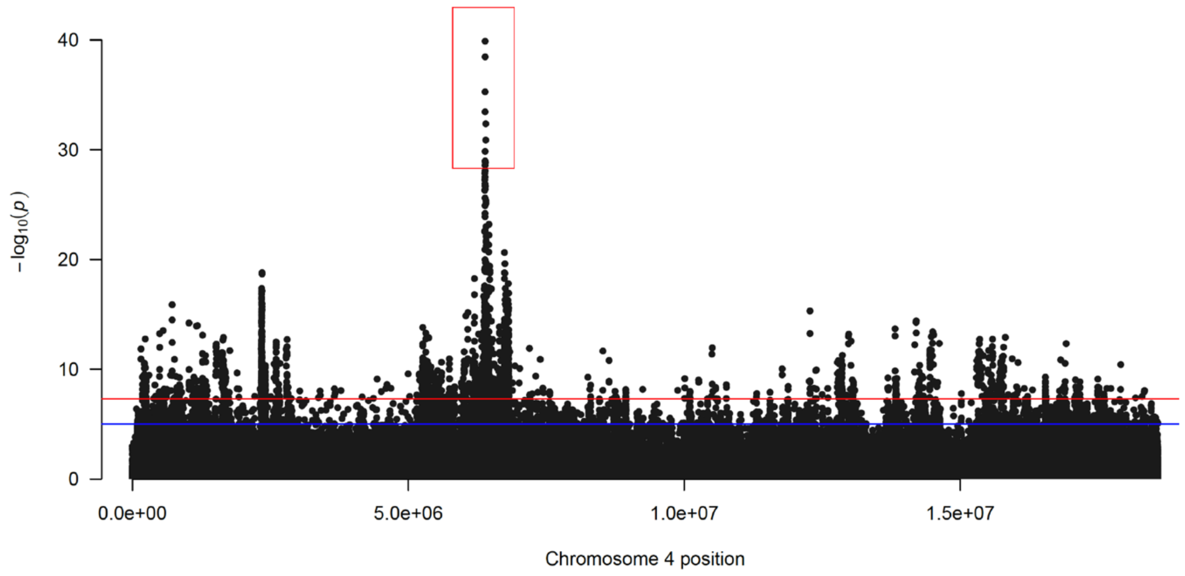

(D) Variance Plots Chromosome 4:

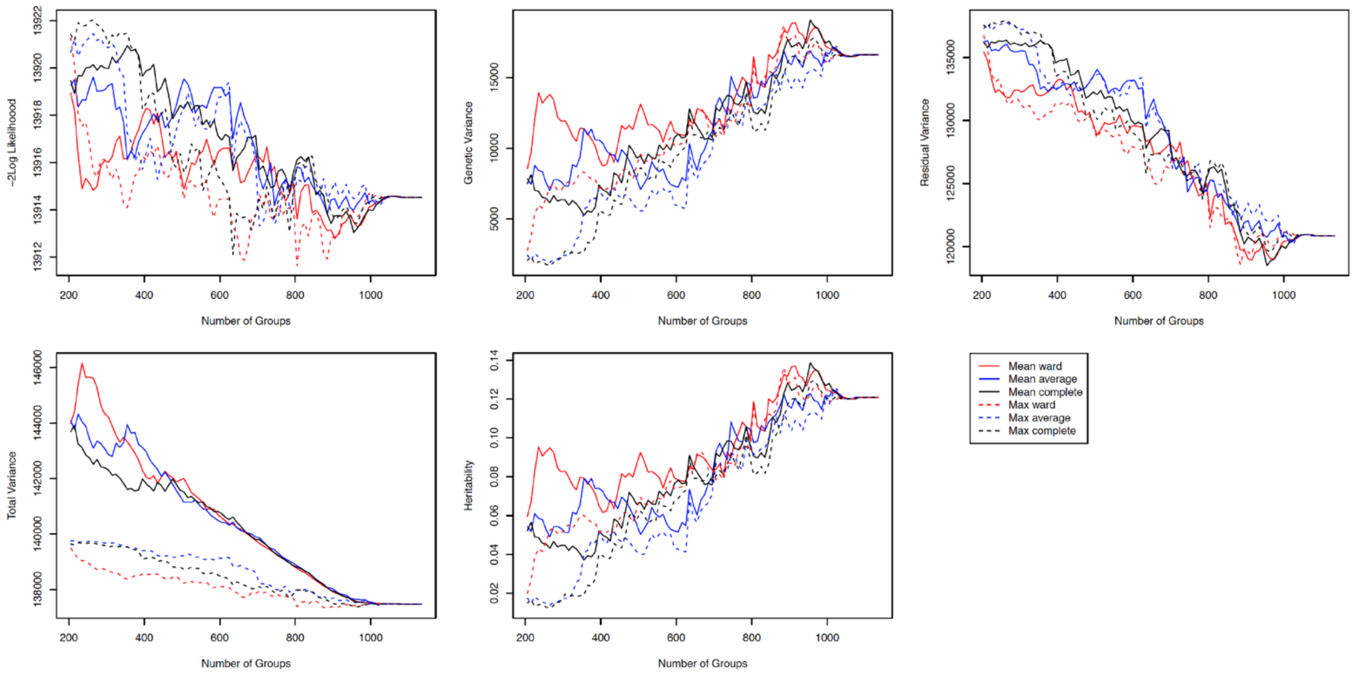

(E) PCA Plots Chromosome 4:

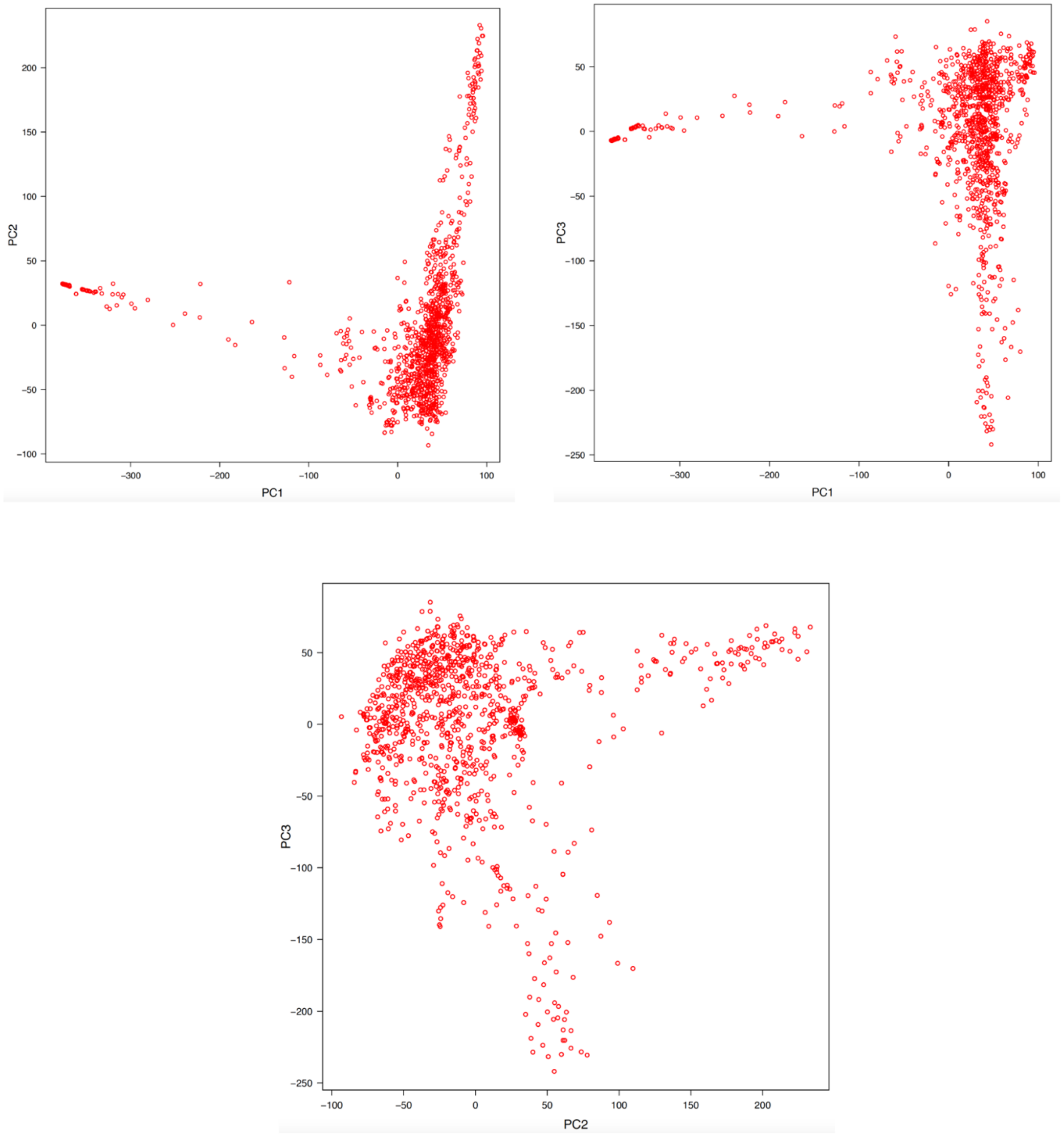

Fig. S4: GWAS analysis output plots.

Figure S5

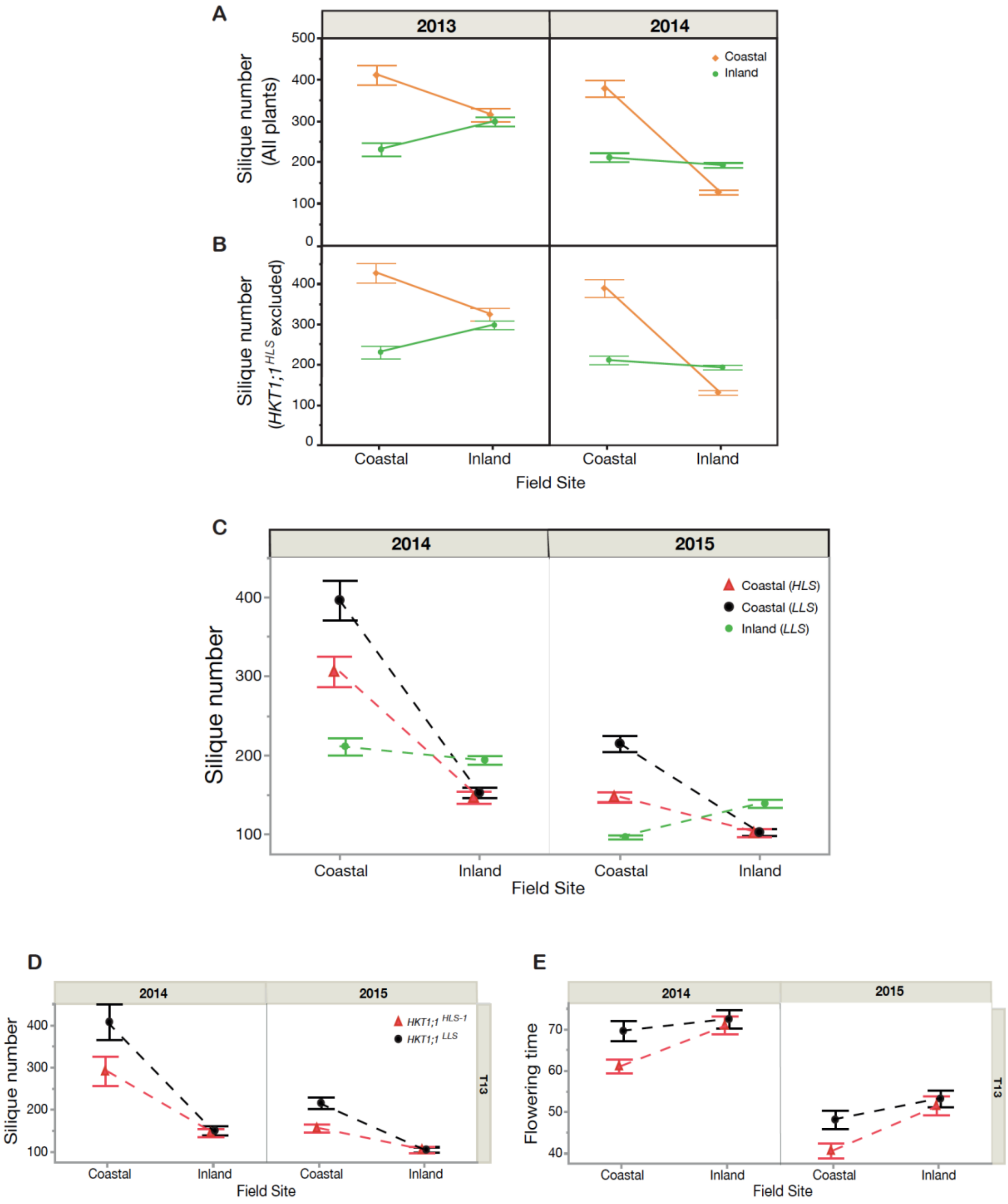

Fig. S5: Fitness (mean ± SE of silique number) of **(A)** the entire set of plants from inland (green) and coastal (orange) demes cultivated at BLA-coastal and SCF-inland common gardens in spring of 2013 and 2014. **(B)** Reanalysis of fitness from the same set of plants (A) excluding the plants harbouring the *HKT1;1*^{HLS} allele version (2013: 12 *HKT1;1*^{HLS} coastal plants; 2014: 22 *HKT1;1*^{HLS} coastal plants). **(C)** Fitness (mean ± SE of silique number) of plants from inland demes (green) and coastal plants harbouring the *HKT1;1*^{LLS} (black) or *HKT1;1*^{HLS} (red) allele version. Reanalysis of fitness **(D)** and flowering time **(E)** from T13 plants excluding the plants potentially harbouring the *HKT1;1*$^{HLS\text{-}2}$ allele version (2014: 4 *HLS* plants; 2015: 5 *HLS* plants excluded).

Figure S6

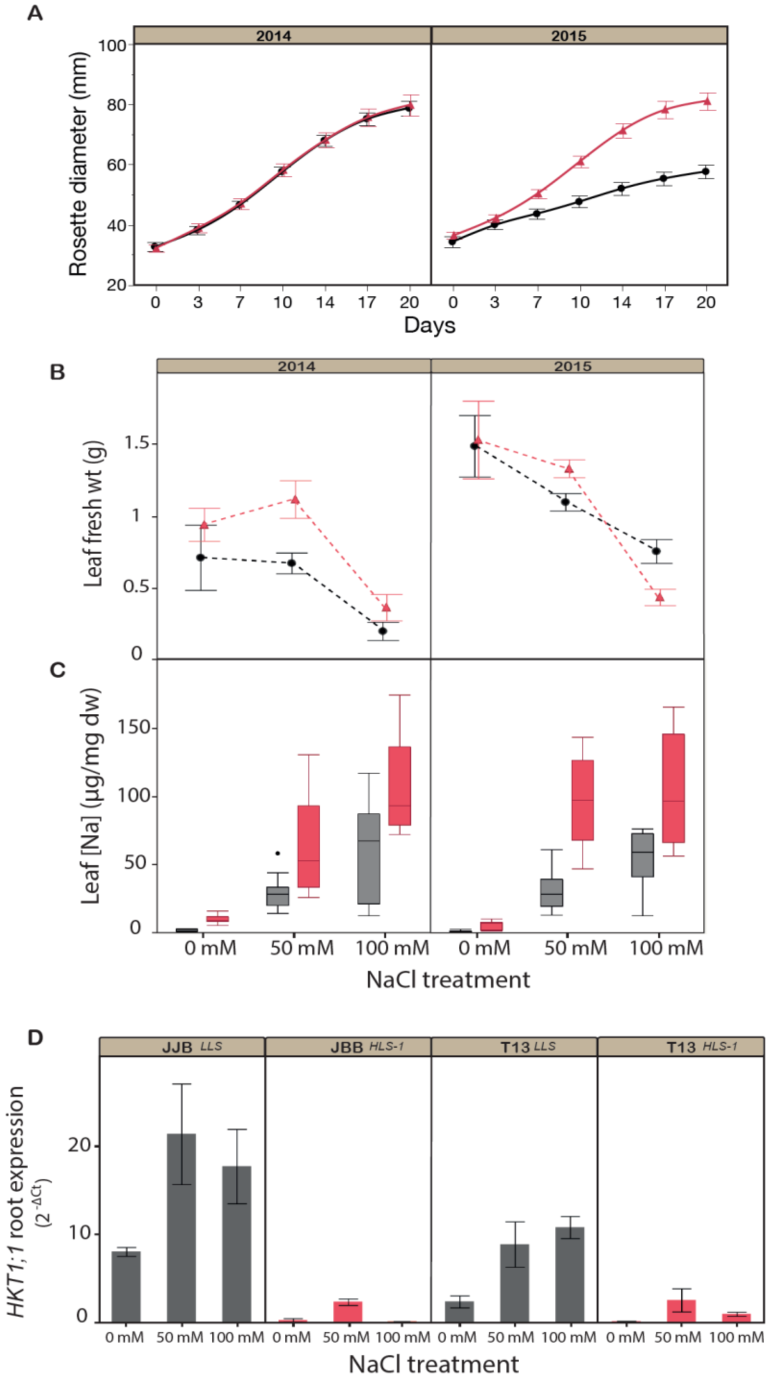

Fig. S6: (A) Growth (rosette diameter, mm) of plants with the $\mathit{HKT1;1}^{LLS}$ (black) and plants with the $\mathit{HKT1;1}^{HLS}$ (red) allele from T13 and JBB demes cultivated in common soil and irrigated with 0 and 50 mM of NaCl for 20 days. Data represents the mean ± SE (n=10 plants per *HKT1;1* allele version and treatment). Rosette fresh weight (g) **(B)** and leaf Na content (µg/g dry weight) **(C)** of plants with the $\mathit{HKT1;1}^{LLS}$ (grey) and plants with the $\mathit{HKT1;1}^{HLS}$ (red) allele from T13 and JBB demes after being exposed to either 0, 50 or 100 mM fo NaCl in hydroponic solution during 2 weeks. Data represents the mean ± SE (n=20 plants per *HKT1;1* allele version, treatment and year). **(D)** Expression profile of *HKT1;1* in roots of three plants from $T13^{LLS}$, JBB^{LLS} (black bars) and T13 ^{HLS}, JBB ^{HLS} (red bars) after being exposed to either 0, 50 or 100 mM of NaCl in hydroponic solution during 2 weeks.

Figure S7

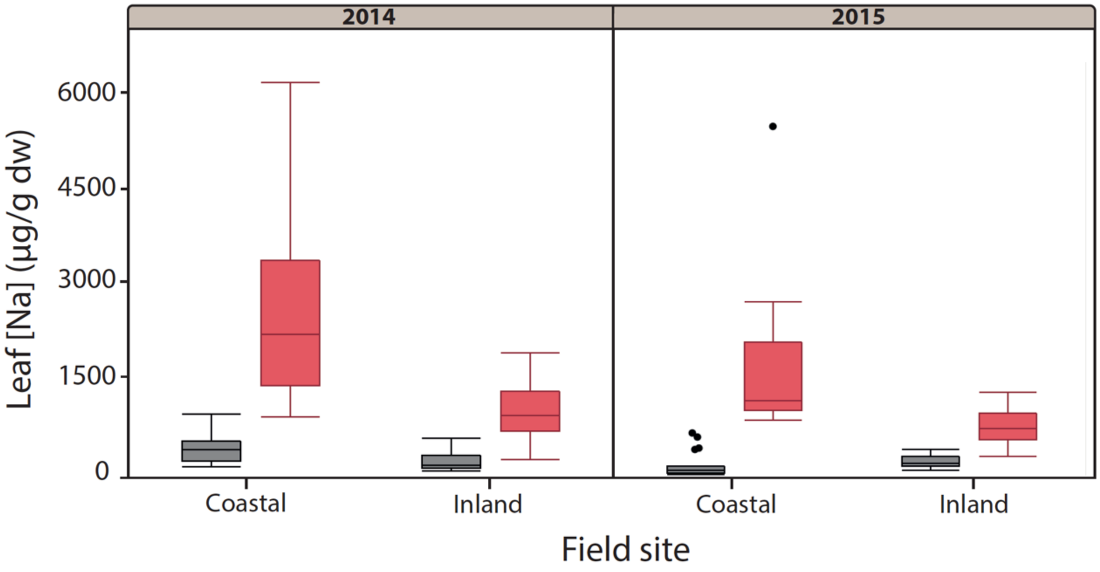

Fig. S7: Leaf Na content (µg/g dry weight) of 20 plants with the *HKT1;1*^{LLS} (black boxes) and 20 plants with the *HKT1;1*^{HLS} (red boxes) allele cultivated at BLA-coastal and SCF-inland fields in spring of 2014 and 2015.

Supplementary Tables

Table S1. Non-synonymous changes in *HKT1;1* coding region between the three versions. Fixation frequency of each SNP substitution among the total individuals of each *HKT1;1* version (*HKT1;1*^{LLS}: n=62, *HKT1;1*$^{HLS-1}$: n=9, *HKT1;1*$^{HLS-2}$: n=6).

Position	Common SNP	Diff SNP	Aa Number	Common Aa	Diff Aa	NS - Aa	Freq *HKT1;1*^{LLS}	Freq *HKT1;1*$^{HLS-1}$	Freq *HKT1;1*$^{HLS-2}$	Fixation in *HKT1;1* versions
Chr4: 6392015	T	G	3	Ile	Arg	I3A	100 % T	100% T	13% T / 88% G	Fix in *HLS-2*
Chr4: 6392037	A	C	10	Lys	Asn	L10C	98% A / 2 % C	100% C	100% A	Fix in *HLS-1*
Chr4: 6392077	G	C	24	Val	Leu	V24L	99% G / 1% C	100% C	11% G 89% C	Fix in *HLS-1 & 2*
Chr4: 6392203	G	C	65	Leu	Val	L65V	99% G / 1% C	100% C	100% G	Fix in *HLS-1*
Chr4: 6392408	G	A	133	Tyr	Cys	Y133A	99% G / 1% A	100% A	100% G	Fix in *HLS-1*
Chr4: 6394436	A	G	143	Gly	Glu	G143E	97% A / 3% G	100% G	100% A	Fix in *HLS-1*
Chr4: 6395577	C	G	476	Phe	Leu	F476L	99% C / 1% G	100% G	2% C / 98% G	Fix in *HLS-1 & 2*
Chr4: 6395624	T	C	492	Val	Ala	V492A	62% T / 38% C	100% C	6% T / 94% C	Fix in *HLS-1 &2*

Table S2. AraGWAS significant hits on At4G10310 (*HKT1;1*) gene (Na23 project) and SNP bases corresponding to our *HKT1;1* alignment.

CHR	BP	Score	Location	Ref	*LLS*	*HLS-1*	*HLS-2*
Chr4	6394966	41.301	1st Intron	G	G	A	G
Chr4	6394436	39.306	Exon 2 (NS-C)	A	A	G	A
Chr4	6390405	35.475	Promoter	G	G	A	G
Chr4	6388539	35.475	Promoter	A	A	G	A
Chr4	6389564	32.424	Promoter	A	A	G	A
Chr4	6392316	24.354	Exon 1 (S-C)	C	C	T	C
Chr4	6392655	24.354	Exon 1 (S-C)	C	C	T	C
Chr4	6392280	24.354	Exon 1 (S-C)	C	C	T	C
Chr4	6392667	24.354	Exon 1 (S-C)	C	C	T	C
Chr4	6388806	23.844	Promoter	G	G	A	G

Table S3. Score, number of observations and corresponding allele, estimate, location and SNP bases corresponding to our *HKT1;1* alignment of the 12 first significant hits.

SNP	Score	maxObs	minObs	maxAllele	minAllele	Estimate 1	Estimate 2	Location	Ref	*LLS*	*HLS-1*	*HLS-2*
Chr4:6392280	39.88	883	50	C	T	-6.91E+02		*HKT1;1* Exon 1	C	C	T	C
Chr4:6392316	38.46	882	51	C	T	-6.74E+02		*HKT1;1* Exon 1	C	C	T	C
Chr4:6391204	35.27	685	180	G	T	-3.80E+02		*HKT1;1* Promoter	T	G	T	T
Chr4:6391286	33.45	755	154	A	C	-3.85E+02		*HKT1;1* Promoter	C	A	C	C
Chr4:6392199	29.84	865	70	C	A	514.40072		*HKT1;1* Exon 1	C	C	A	C
Chr4:6392067	28.97	703	89 / 76	C	A / T	-3.26E+02		*HKT1;1* Exon 1	A	C	T	A
Chr4:6395592	28.83	797	80	A	G	-4.72E+02		*HKT1;1* Exon 3	A	A	G	G
Chr4:6392077	28.56	714	160	G	C	353.46149	-5.58E+02	*HKT1;1* Exon 1	C	G	C	C
Chr4:6392079	28.07	738	139	A	T	-3.72E+02		*HKT1;1* Exon 1	T	A	T	T
Chr4:6388578	27.85	541	136	G	A	390.47711		*HKT1;1* Promoter	G	G	T	T

Table S4. ANOVA (Fisher test) of fitness from inland and coastal *A. thaliana* demes cultivated at BLA-coastal and SCF-inland common gardens in spring of 2013 and 2014. Results including all plants and reanalysis excluding the plants harbouring the *HKT1;1* ^{HLS} allele version.

	Field Site	Year	Location	N	Mean	Std Dev	F Ratio	Prob > F
All plants	BLA	2013	Coastal	83	410.58	218.9	38.6416	< 0.001
			Inland	80	230.69	140.67		
		2014	Coastal	100	378.1	205.4	51.5591	< 0.001
			Inland	100	211.14	108.97		
	SCF	2013	Coastal	85	314.15	146.73	0.7322	0.3933
			Inland	93	297.85	105.77		
		2014	Coastal	100	127.02	57.59	65.5536	< 0.001
			Inland	100	192.86	57.41		
HKT1;1 ^{HLS} excluded	BLA	2013	Coastal	76	426.83	212.84	46.544	< 0.001
			Inland	80	230.69	140.67		
		2014	Coastal	88	389.33	202.66	58.2106	< 0.001
			Inland	100	211.14	108.97		
	SCF	2013	Coastal	80	324.18	144.49	1.9027	0.1696
			Inland	93	297.85	105.77		
		2014	Coastal	90	130.06	57.38	56.7172	< 0.001
			Inland	100	192.86	57.41		

Table S5. Quality control parameters of DNA library preparation for the *de novo* genome assembly of three *A. thaliana* samples.

Input sample QC			Final library QC			
Sample	Input QuBit [] (ng/µl)	Total input in GEM (ng)	QuBit [] (ng/µl)	Average fragment size (bp)	Molarity (nM)	Index used
S1 (T11)	0.456	0.57	1.15	638	2.82	A3
S2 (JBB)	0.592	0.62	5.54	659	12.95	B3
S12 (PA10)	0.512	0.64	7.02	594	18.21	B5

Table S6. Assembly metrics of three *A. thaliana* genome assemblies using 10x Genomics Chromium platform and Supernova assembler.

Sample	Deme	Allele	Sequencing	N50	Assembly size	Coverage
S1	T11	*HKT1;1* ^{LLS}	150PE	20 kb	60 Mb	37x
S2	JBB	*HKT1;1* $^{HLS-1}$	150PE	240 kb	100 Mb	64x
S12	PA10	*HKT1;1* ^{LLS}	250PE	1015 kb	110 Mb	63x

Other supplementary material

Additional data Dataset S1 (separate file)

Dataset S1. Sequencing sample information: name, deme of origin, GPS coordinates, location and *HKT1;1* allelic variant.

Additional data Dataset S2 (separate file)

Dataset S2. Leaf sodium (mg/kg dw) concentrations of 1119 *A. thaliana* world-wide accessions.

References

1. Martin M (2011) Cutadapt removes adapter sequences from high-throughput sequencing reads. EMBnet J 17(1): 10-12.

2. Bolger AM, Lohse M, Usadel B (2014) Trimmomatic: a flexible trimmer for Illumina sequence data. Bioinformatics: 1–7.

3. McKenna A, Hanna M, Banks E, et al. (2010) The Genome Analysis Toolkit: a MapReduce framework for analysing next-generation DNA sequencing data. Genome Res 20 (9): 1297-303.

4. Lipka AE, Tian F, Wang Q, Peiffer J, Li M, Bradbury PJ, Gore MA, Buckler ES, Zhang Z (2012) GAPIT: genome association and prediction integrated tool. Bioinformatics 28: 2397–9.

5. VanRaden PM (2008) Efficient methods to compute genomic predictions. J Dairy Sci 91: 4414–4423

6. Bradbury PJ, Zhang Z, Kroon DE, Casstevens TM, Ramdoss Y, Buckler ES (2007). TASSEL: software for association mapping of complex traits in diverse samples. Bioinformatics 23: 2633–5.
